# Virtual energy shortage risk to trade network in China

**DOI:** 10.1016/j.fmre.2025.11.005

**Published:** 2025-11-13

**Authors:** Hanlei Wang, Hui Li, Yulei Xie, Frederick Kwame Yeboah, Zhiyao Ding, Gengyuan Liu, Yixuan Wang

**Affiliations:** a School of Ecology, Environment and Resources, Guangdong University of Technology, Guangzhou 510006, China; b School of Environment, Beijing Normal University, Beijing 100875, China; c College of Water Conservancy and Civil Engineering, Inner Mongolia Agricultural University, Hohhot 010018, China

**Keywords:** Energy shortage risk, Cascading effects, Input-output analysis, Trade network, China

## Abstract

Local energy shortages can directly lead to economic output losses in energy-intensive sectors, known as local energy shortage risk (LESR). Additionally, the impacts of local energy shortages can propagate through supply chains, creating cascading effects that induce potential indirect economic losses in external regions and sectors, termed virtual energy shortage risk (VESR). This study quantifies the propagation of VESR across China’s regional trade network from 2012 to 2017, uncovering the systemic risk posed by local energy shortages. The results show that VESR accounted for a substantial proportion (41.0–43.9%) of China’s energy shortage risk (ESR, the sum of LESR and VESR) during the study period, highlighting the significance of indirect impacts. LESR and VESR exhibit notable geographic separation. For instance, Tibet experienced no LESR but is exposed to VESR. VESR exporters are concentrated in energy-intensive sectors within energy-scarce regions, such as “Metal Smelting and Pressing” in Hebei and Henan. In contrast, VESR importers are more widely distributed; sectors such as “Agriculture Services” and “Coal Mining and Dressing” in Beijing, experience VESR imports exceeding 50% of their ESR, indicating high external risk exposure. These findings highlight the need for inter-regional coordination in energy resource management and provide risk mitigation pathways for VESR importers (e.g., diversifying supply sources and strengthening energy supply technology cooperation with upstream regions).

## Highlights

• Local energy shortage risk cascades through trade, creating virtual risk patterns.
• Virtual energy shortage risk propagation in China is measured based on the MRIO.
• Virtual energy shortage risk constitute 41–44% of total risks during 2012–2017.
• Developing virtual risk mitigation strategies strengthens trade network resilience.

## Graphical abstract

**Figure:**
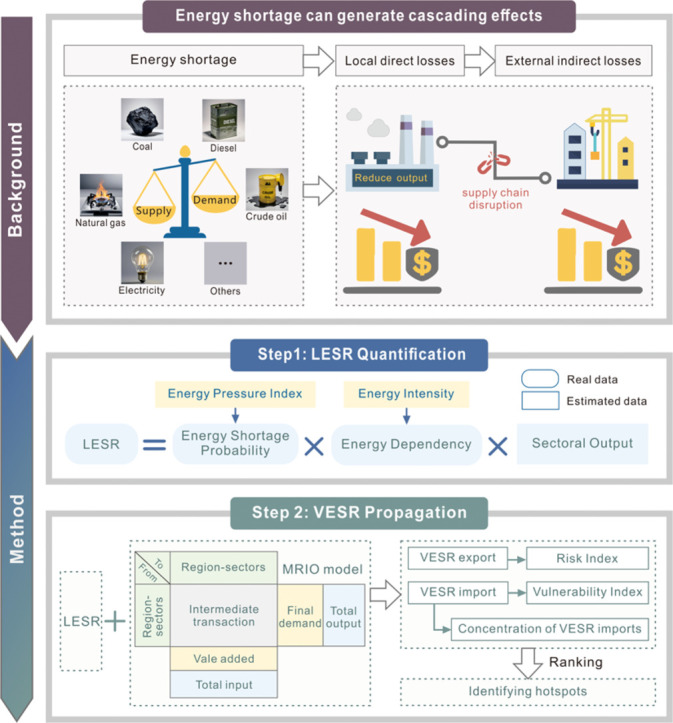

## 1 Introduction

Energy serves as the lifeblood of the global economy, playing an indispensable role in modern society [1]. With the acceleration of industrialization and rapid economic development worldwide, energy demand has increased significantly [2]. The substantial gap between energy supply and demand has caused severe energy shortages in many countries and regions, posing significant challenges to economic development and social stability [3,4]. These challenges are more pronounced in large developing economies such as China. As the world’s largest developing country and second-largest economy, China’s energy consumption has grown exponentially over the past three decades [5,6]. However, domestic energy resource endowment in China remains relatively limited. Per capita coal and hydropower resources are approximately 50% of the global average, while per capita oil and natural gas resources represent only about 7% [7]. Ensuring energy security has emerged as a critical foundation for achieving sustainable socioeconomic development in China [8,9].

Although energy resources are primarily managed at the local or regional level, energy allocation is increasingly influenced by expanding interregional economic linkages. These linkages manifest in two primary forms of energy flows: direct physical flows of energy commodities and embodied energy flows associated with traded goods and services [10]. The direct physical transfer refers to the movement of energy resources, such as coal, gas, or electricity, from resource-rich areas to regions with concentrated demand [11,12]. In China, this process is mainly driven by diverse infrastructure projects such as the West-to-East Gas Transmission and West-to-East Power Transmission [13,14]. The flow of embodied energy refers to the transfer of cumulative energy inputs embedded in goods and services produced and traded between regions [15]. This virtual flow is crucial for assigning energy consumption responsibility and carbon emissions [16]. The geographical separation of production and consumption leads to spatial redistribution of energy use, creating patterns where producers bear environmental costs while consumers benefit [17]. For instance, coal-producing regions bear environmental costs, whereas urban consumers benefit from the energy embedded in traded goods. Many studies employing multi-regional input-output (MRIO) models have systematically quantified these embodied energy flows, showing that external consumption is a key driver of local energy use and a factor reshaping regional energy balances [15,18,19].

As interregional energy flows become increasingly complex, the propagation of energy shortage risk across regions has intensified. Energy systems and economic activities are deeply interconnected: while economic development shapes patterns of energy production and consumption, energy shortages constrain industrial output and economic stability [20,21]. Energy shortages initially impact local production, particularly in energy-intensive sectors, resulting in local energy shortage risk (LESR) [22]. These disruptions can further propagate through interconnected trade networks, generating cascading effects that extend well beyond the originating region [[23], [24], [25]]. Disruptions at critical supply chain nodes may cause substantial indirect losses in distant areas, irrespective of their own local energy adequacy [26,27]. For example, if an energy shortage constrains the output of an upstream sector in one region, downstream sectors in other regions that depend on its intermediate inputs may experience supply disruptions and corresponding indirect economic losses. These indirect economic losses stemming from upstream energy shortages can be defined as virtual energy shortage risk (VESR). VESR can be substantial; for instance, studies have shown that power system failures can trigger widespread economic disturbances across interregional supply chains [28,29]. In China, the management of local energy resources is becoming increasingly intertwined with broader trade systems [30,31]. Therefore, it has become essential for industrial and commercial policies to explicitly account for these interregional linkages to effectively identify and mitigate VESR within the national trade network.

This study focuses on identifying and characterizing the propagation patterns of VESR across China’s regions and sectors from 2012 to 2017. First, a novel monetized metric is developed to assess LESR at the region-sector level (i.e., a specific sector within a given region), integrating comprehensive data on regional energy consumption, supply, and economic output. We then systematically examine how LESR propagate through China’s interconnected trade network and give rise to VESR in other regions and sectors based on the MRIO model. While previous research examined only direct economic losses from energy shortages, the current framework tracks the cross-regional propagation of these risks. By identifying critical VESR exporters and importers, our analysis provides targeted insights to enhance the resilience of China’s trade network against energy shortages.

## 2 Methodology

**Fig. 1:**
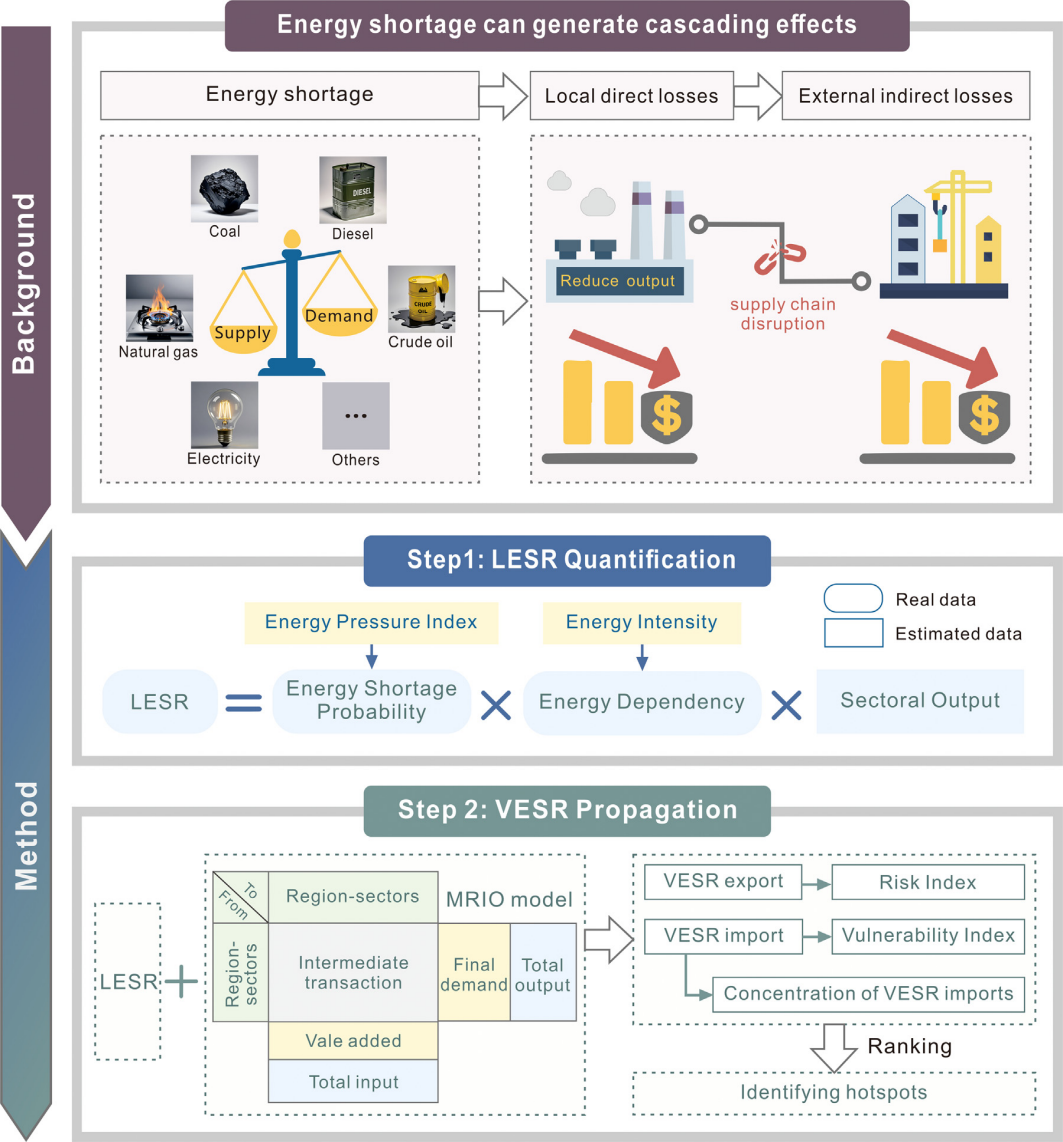
**Graphical illustration of the analytical framework of this study.** Note: LESR: local energy shortage risk; VESR: virtual energy shortage risk.

This study defines the energy shortage risk (ESR) of a region-sector as the potential economic losses (measured in monetary units) induced by energy shortages. For a region-sector affected by energy shortage, its economic losses can be categorized into two components: (1) direct losses attributed to its local energy shortage (i.e., LESR); and (2) indirect losses stemming from the cascading effects of external energy shortages (i.e., VESR). LESR and VESR are defined as two non-overlapping components, which avoids double-counting. A comprehensive framework has been developed to assess both LESR and VESR (Fig. 1).

It should be noted that the results related to ESR are relative values rather than absolute values. In other words, the ESR values do not represent actual economic loss amounts but rather potential relative economic loss quantities. The objective of this study is not to achieve a precise monetization assessment of the impact of energy shortage, but to identify hotspots of LESR and propagation of VESR by comparing the relative magnitudes of these values.

### 2.1 LESR measurement

The LESR of a region-sector is the potential direct economic loss resulting from insufficient local energy supply. A region-sector whose economic activities are dependent on energy may experience economic loss if it cannot obtain enough energy to meet its needs. The magnitude of these losses depends on the level of reliance on energy for production. The connection between LESR, the energy shortage probability (EP), and the energy dependence (ED) can be estimated as

(1) $$LESR_{m,i}=EP_{i}\times ED_{m,i}\times x_{m,i}$$

Where $LESR_{m,i}$ is the LESR of sector m in region i; $EP_{i}$ represents the energy shortage probability of region i; $ED_{m,i}$ indicates the energy dependency level of sector m in region i, evaluating the percentage loss in economic output resulting from a 1% energy shortage, and $x_{m,i}$ represents the total output of sector m in region i. Given the absence of direct statistical data on EP and ED, values were extrapolated based on associated variables as elaborated below.

#### 2.1.1 Energy shortage probability (EP)

The indicator EP is bounded within the interval [0,1] and is utilized to quantify the probability of energy shortage within a region. The energy pressure index (EPI) is defined as the ratio of energy consumption (CE) to energy supply (SE) and is estimated as follows:

(2) $$EPI_{i}=\frac{\sum_{n=1}^{g}CE_{i}^{k}}{\sum_{n=1}^{g}SE_{i}^{k}}=\frac{\sum_{n=1}^{g}CE_{i}^{k}}{\sum_{n=1}^{g}\left(PE_{i}^{k}+IME_{i}^{k}-EXE_{i}^{k}\right)}$$

Where $EPI_{i}$ represents the energy pressure index of region i. $SE_{i}^{k}$ and $CE_{i}^{k}$ denote the supply and consumption, respectively, of the kth type of energy in region i. g represents the total number of energy types included in this study. $SE_{i}^{k}$ is the sum of the local energy production $\left(PE_{i}^{k}\right)$ and the energy imports $\left(IME_{i}^{k}\right)$, minus the energy exports$\left(EXE_{i}^{k}\right)$. We employ a function that converts EPI into EP:

(3) $$EP_{i}=f_{EP}\left(EPI_{i};\sigma \right)=E\left(Y_{i}\right)$$

(4) $$Y_{i}=\left\{ \begin{array}{c} 0,\;\;X_{i}\geq 1\\ 1-X_{i},\;X_{i}<1 \end{array}\right.$$

(5) $$X_{i}\;∼\;\text{Lognormal}\left(\mu_{i},\;\sigma^{2}\right)$$

(6) $$\mu_{i}=\text{log}\frac{1}{EPI_{i}}$$

In (3), (4), (5), (6) $EP_{i}$ (dimensionless) denotes the energy shortage probability of region i. The function $f_{EP}$ is utilized to convert $EPI_{i}$ into $EP_{i}$, equivalent to the expected value of the variable $Y_{i}$ (dimensionless). $Y_{i}$ represents the energy shortage within region i at each spatiotemporal unit. $X_{i}$ is a random variable representing the ratio of the energy supply and consumption within region i at each spatiotemporal unit. Hence, when $X_{i}\geq 1$, $Y_{i}=0$; conversely, when $X_{i}<1$, $Y_{i}=1-X_{i}$. The random variable $X_{i}$ follows a log-normal distribution, with a variance $\mu_{i}$ equal to the logarithm of $\frac{1}{EPI_{i}}$. The parameter σ represents the standard deviation. A larger σ implies a greater difference in EP between a high-EPI and a low-EPI region.

#### 2.1.2 Energy dependency (ED)

Energy dependency (ED), defined as the percentage loss in economic output of a region-sector due to a 1% reduction in energy consumption, ranges from 0 to 1. A value of 1 implies that energy is completely irreplaceable for that region-sector’s economic activities, leading to a proportional decline in economic output as the energy supply decreases. We use energy intensity (EI), defined as the energy consumption per unit of economic output, to assess a region-sector’s ED. The EI of a region-sector can be converted into its ED as follows:

(7) $$ED_{m,i}=f_{ED}\left(EI_{m,i};\;\alpha \right)=\frac{1}{1+exp\left(-\alpha EI_{m,i}\right)\left(\frac{1}{0.001}-1\right)}$$

(8) $$EI_{m,i}=\frac{CE_{m,i}}{x_{m,i}}$$

In Eqs. 7 and 8, $ED_{m,i}$ (dimensionless) represents the energy dependency level of sector m in region i; $EI_{m,i}$ is the energy intensity of sector m in region i, calculated as the ratio of the region-sector’s energy consumption ($CE_{m,i}$) to its total output ($x_{m,i}$). α is the truncation parameter for $ED_{m,i}$, which controls the cutoff value of EI above which ED rapidly approaches 1.

### 2.2 MRIO model

We reveal the cascading effects of LESR by tracing VESR based on the MRIO model. The MRIO model enables the analysis of economic transactions at the regional and sectoral level, as well as inter-regional interactions [32]. Within the MRIO table, the total input of each sector is equal to the sum of its intermediate inputs and value-added or primary inputs, which is referred to as column balance, as shown in Eq. 9:

(9) x=eZ+v

Suppose an economic system consists of n production sectors. The 1×n row vector v represents the initial input of each sector, the n×n matrix Z represents inter-sectoral product transactions, the n×1column vector x represents the total input of each sector, and all elements of the n×1column vector e are equal to 1.

Let B be an n×n matrix defined as $B={\left(\hat{x}\right)}^{1}\times Z$, where the symbol “^” signifies the diagonalization of the vector, and the symbol “−1” represents matrix inversion. The matrix B is the direct output coefficient matrix, with its elements $b_{ij}$ denoting the direct output of sector j resulting from the production of one unit of output from sector i. Accordingly, Eq. 9 can be converted as

(10) $$x=v{\left(I-B\right)}^{-1}$$

Eq. 10 establishes the correlation between total output x and initial input v. The matrix ${\left(I-B\right)}^{-1}$ is the Ghosh inverse matrix [[32], [33], [34]], with its elements $g_{ij}$ denoting the cumulative (including direct and indirect) output of sector j resulting from the production of one unit of output from sector i. We evaluate the VESR propagation by adopting the Ghosh inverse matrix, as illustrated in the subsequent equation.

(11) $$\Delta E_{m,i}^{n,j}=diag\left(LESR\right)\times {\left(I-B\right)}^{-1}$$

In Eq. 11,ΔE (measured in monetary units) represents the matrix of VESR propagation. The element $\Delta E_{m,i}^{n,j}$ indicates the VESR from sector m in region i to sector n in region j, quantifying the cascading effect of energy shortage of sector m in region i(donor) on the downstream region-sector (recipient). The expression “diag (*LESR*)” refers to the diagonalization of the vector LESR. By excluding the diagonal elements of matrix ΔE and performing row-wise and column-wise summation, the VESR export and import for each region-sector, termed $VESR_{i}^{EX}$ and $VESR_{i}^{IM}$, can be obtained respectively:

(12) $$VESR_{i}^{EX}=\sum_{i\neq j}n_{ij}$$

(13) $$VESR_{i}^{IM}=\sum_{j\neq i}n_{ji}$$

To mitigate the impact of economic scale on VESR, the VESR imports and exports of each region (or region-sector) are normalized by their total output. A risk index (RI) is defined to quantify the VESR export per unit of output, and a vulnerability index (VI) to assess the VESR import per unit of output. A higher RI indicates that a region (or region-sector) poses a greater risk to the trade network due to its higher VESR exports per unit of output. A higher VI reflects greater vulnerability to external energy shortages. The estimation of RI and VI is depicted in Eqs. 14 and 15:

(14) $$RI_{i}=\frac{VESR_{i}^{EX}}{x_{i}}$$

(15) $$VI_{i}=\frac{VESR_{i}^{IM}}{x_{i}}$$

Where $RI_{i}$ (dimensionless) represents the risk index of region (or region-sector) i, $VI_{i}\;$(dimensionless) denotes the vulnerability index of region (or region-sector) i and $x_{i}$ represents the total output of region (or region-sector) i.

### 2.3 Herfindahl index

Using the Herfindahl-Hirschman Index (HHI) to measure the concentration of VESR imports within a specific region-sector helps assess its vulnerability to VESR imports [35,36]. HHI values below 0.01 indicate a highly dispersed set of upstream suppliers, while values between 0.01 and 0.15 suggest a relatively balanced distribution. Values ranging from 0.15 to 0.25 indicate a moderately concentrated supplier base, and values exceeding 0.25 signify a high concentration of suppliers [37]. A region-sector with a higher HHI has a more concentrated source of VESR imports and is more sensitive to energy shortages in upstream sectors and vulnerable to indirect economic losses. HHI is estimated as

(16) $$HHI_{i}=\sum_{j\neq i}{\left(\frac{n_{ji}}{\sum_{j\neq i}n_{ji}}\right)}^{2}$$

## 3 Data sources

The study requires three types of data: national MRIO tables, sector-specific energy consumption data for each region, and regional energy supply data. The year 2017 was selected as the baseline, primarily because it corresponds to the latest update of the input-output table at the time of this research. To capture temporal dynamics in both LESR and VESR, supplementary analyses were also conducted for 2012 and 2015.

The Chinese MRIO tables, which cover 31 regions with 42 sectors in each region, were obtained from the China Emission Accounts and Datasets (CEADs) [38]. Due to data availability constraints, Taiwan, Hong Kong, and Macao were excluded from the analytical scope. For inter-temporal comparability, current-price MRIO data in 2012 and 2015 were converted to 2017 constant prices using the “convert-first-then-deflate” method [39].

Energy data encompassed ten categories derived from the China Energy Statistical Yearbook, including primary energy (coal, crude oil, and natural gas) and secondary energy (coke, gasoline, kerosene, diesel, fuel oil, liquefied petroleum gas, and electricity). Regional sector-specific energy consumption data were obtained from the provincial energy inventory in CEADs [[40], [41], [42], [43]] and the China Energy Statistical Yearbook [44]. These datasets explicitly record energy use in agricultural and industrial sectors at the region-sector level, whereas energy consumption data for service sectors are only reported as regional aggregates. To align with the sectoral resolution of the MRIO model, we allocated the energy consumption from original statistics to each service region-sector based on their shares in total output.

Regional energy supply data was calculated by combining local energy production and energy imports, then subtracting energy exports. Details on energy production, imports, and exports were sourced from the Chinese Energy Statistical Yearbook. All energy values were converted into standard coal units using conversion factors (see Supplementary Information (SI) Table S1) from the China Energy Statistical Yearbook.

## 4 Results

### 4.1 Energy shortage probability (EP) and energy dependency (ED)

**Fig. 2:**
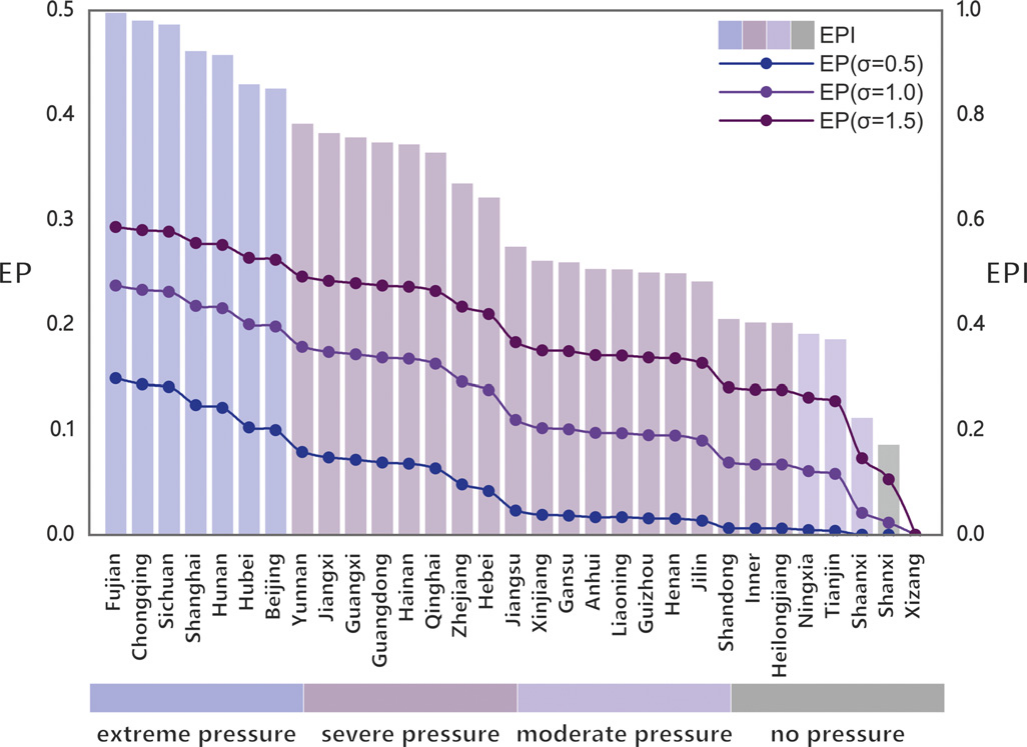
**The EPI and corresponding EP of each region in China under different σ values.** Note: The bar chart uses different colors to represent various energy pressure levels. Regions are arranged in descending order based on their EPI values.

EP represents the probability of energy shortage within a region, ranging from 0 to 1. Due to limited data on EP, we hypothesize that regions with high EPI values are more likely to face the risk associated with energy shortages. By applying a transformation function to convert EPI values to EP values, Fig. 2 presents the EPI levels for each region and the EP values computed at various values of σ (Eq. 3). The EPI is categorized into four levels: “no energy pressure” (EPI < 0.2), “moderate energy pressure” (0.2 < EPI < 0.4), “severe energy pressure” (0.4 < EPI < 0.8), and “extreme energy pressure” (EPI > 0.8). Regions such as Fujian, Chongqing, Sichuan, and Shanghai, with higher EPI values, are identified as more vulnerable to severe energy shortages. We observe that when σ is set to 1, the EP values remain consistently above 0.01 in regions experiencing moderate energy pressure (0.2 < EPI < 0.4). Conversely, when σ is adjusted to 0.5, the EP values tend to approach zero in regions with moderate energy pressure (0.2 < EPI < 0.4). Notably, with σ increased to 1.5, the EP values become significantly pronounced across nearly all regions, including those with no energy pressure (EPI < 0.2). This justifies the selection of σ = 1 as the optimal parameter for this study. Additionally, we conduct a sensitivity analysis using values of σ ranging from 0.5 to 1.5 to assess the robustness of the results.

The EI and corresponding ED for all region-sectors under the parameters of α = 1.5, α = 2, and α = 2.5 are illustrated in Fig. S1. Region-sectors with high EI values generally exhibit high ED levels, with ED values capped at 1. The transformation function from EI to ED, as defined in Eq. 7, has been carefully chosen to reflect this relationship. When α is set to 2, approximately 145 region-sectors display high ED values (greater than 0.999), while 236 region-sectors show low ED values (close to 0.001). Changing α to 1.5 or 2.5 results in fewer or more region-sectors being categorized as having high ED. α = 2 was selected as the optimal parameter for transformation. We also calculated the results for α values ranging from 1.5 to 2.5 in the sensitivity analysis. As demonstrated in Fig. S1b, the shift from energy intensity (EI) to energy dependency (ED) is continuous. However, once EI surpasses 0.69 tce per million RMB, ED reaches its maximum value of 1, indicating high energy dependency. For example, although the entertainment sector in Heilongjiang has a much lower EI (0.69 tce per million RMB) compared to the coal mining and dressing sector in Hebei (3.34 tce per million RMB), both sectors exhibit an ED value of 1. Despite substantial differences in energy intensity, both sectors have reached a saturation point of energy reliance.

### 4.2 LESR quantification

China’s regional LESR exhibits significant spatial disparities. In 2017, high-LESR regions included Sichuan (247 billion RMB), Hebei (189 billion RMB), Jiangsu (136 billion RMB), Hunan (134 billion RMB), and Hubei (118 billion RMB), while Tibet (0 billion RMB) and Shaanxi (0.1 billion RMB) had minimal LESR. These disparities stem from supply-side and demand-side factors. Supply-side drivers include uneven local energy resource endowments and differential energy infrastructure development, which directly affect energy production and distribution capacities. Demand-side drivers involve variations in industrialization levels and sectoral compositions (energy-intensive vs. non-energy-intensive), shaping regional energy needs. Sichuan’s highest LESR mainly results from over 80% reliance on hydropower-rendering it vulnerable to climate variability (e.g., severe droughts). This risk is amplified by surging power demand (from economic growth and heatwave cooling needs) and mandatory electricity exports amid local shortages. In contrast, Tibet’s low LESR is attributed to abundant renewable resources (theoretical hydropower capacity: ∼600 billion kWh; annual sunshine >3000 h, solar radiation 7000 MJ/m² [45,46] and expanded renewable infrastructure (hydroelectric/ photovoltaic stations), which boost self-sufficiency. Its low industrialization (lack of energy-intensive sectors) also limits energy demand, resulting in high energy security and low LESR.

**Fig. 3:**
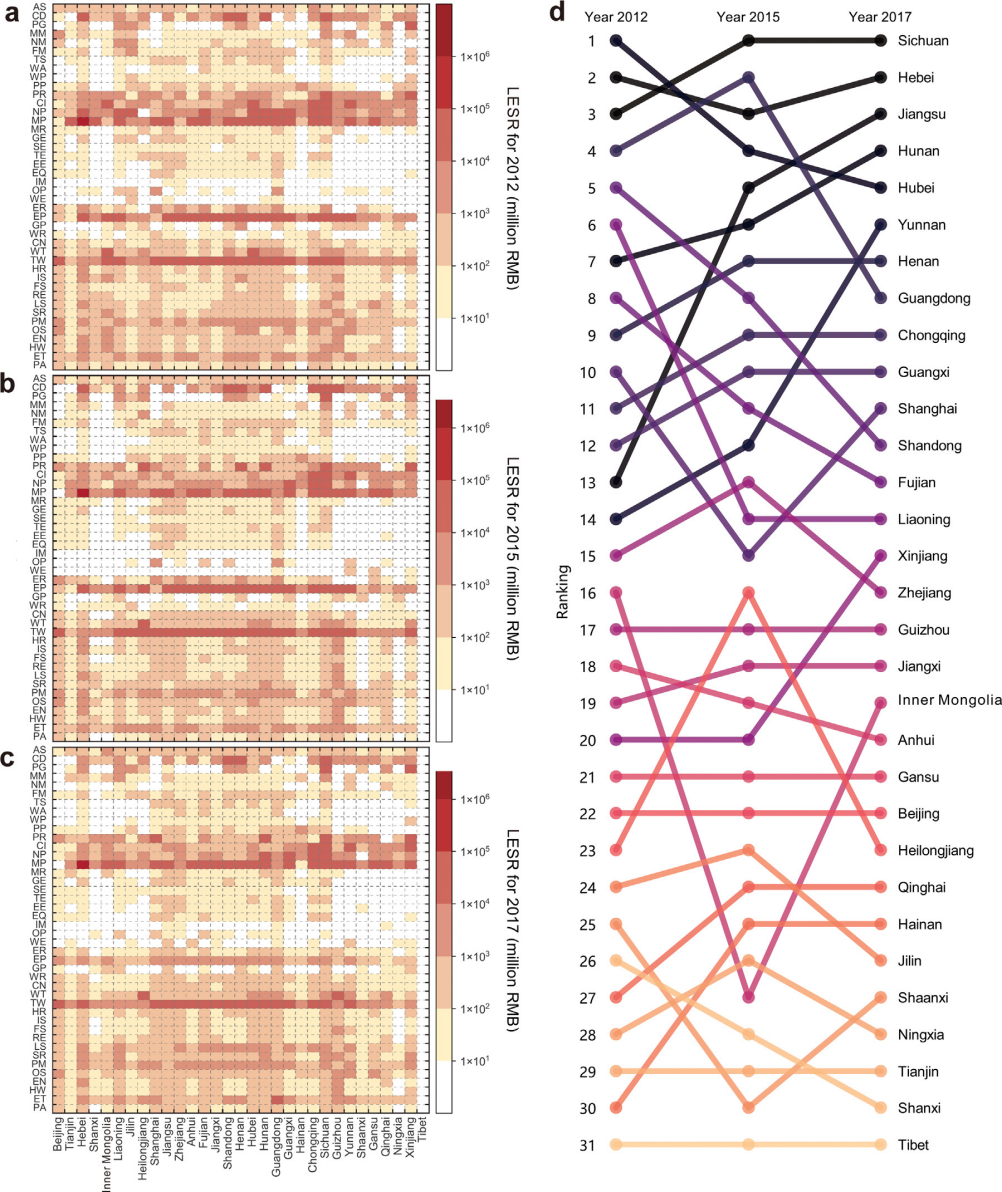
The LESR of each region-sector in China for the years 2012 (a), 2015 (b), and 2017 (c), with temporal evolution of regional rankings regarding LESR (d). Note: The columns in (a–c) represent the respective sectors. The full names of these sectors are provided in SI Table S2.

Region-sectors with high LESR in 2017 include the “Metal Smelting and Pressing” sector in Hebei (141 billion RMB), Jiangsu (89 billion RMB), and Sichuan (61 billion RMB), and the “Transportation and Warehousing” sector in Sichuan (64 billion RMB) and Guangdong (59 billion RMB) (Fig. 3c). The hotspots with high LESR are mainly concentrated in the “Metal Smelting and Pressing” and “Transportation and Warehousing” sectors. These region-sectors are characterized by high EI, with production processes heavily reliant on energy resource inputs. For instance, the EI of the “Metal Smelting and Pressing” and “Transportation and Warehousing” sectors reached 20.6 tce and 35.3 tce per million RMB, respectively, significantly higher than that of other industrial sectors. This resulted in high ED, rendering them more sensitive to local energy shortages. Conversely, certain services (e.g., Public Administration, 2.1 tce per million RMB) and industrial sectors (e.g., Instruments and Meters, 1.0 tce per million RMB) exhibited relatively low EI, leading to low LESR.

We also examined the temporal evolution of LESR at both region and sector levels from 2012 to 2017. During this period, China’s total LESR decreased from 3.1 trillion RMB in 2012 to 2.3 trillion RMB in 2017. This overall downward trend was primarily driven by the reduction in China’s overall energy intensity, indicating enhanced energy efficiency. However, several regions exhibited increasing LESR trends, particularly in Jiangsu, Hainan, Yunnan, Qinghai, and Xinjiang, which showed significant rises in both the absolute values and rankings (Fig. 3d). At the region-sector level, 650 region-sectors experienced LESR increases, especially the “Metal Smelting and Pressing” sectors in Jiangsu and Inner Mongolia, the “Transportation and Warehousing” sector in Sichuan, and the “Wholesale and Retail Trade” sector in Guizhou (Fig. 3a–c). For these regions and sectors, improving energy efficiency and optimizing the energy supply structure are required to reduce LESR.

### 4.3 VESR propagation

#### 4.3.1 Regional level

Between 2012 and 2017, China’s VESR decreased by 33.3%, from 2.4 trillion RMB to 1.6 trillion RMB However, the share of VESR in the ESR (i.e., the combined VESR and LESR) experienced a slight reduction (2.9%) (Fig. S2). This indicates the persistence and significance of VESR in energy shortage risk composition. Understanding its propagation characteristics is crucial for enhancing trade network resilience.

**Fig. 4:**
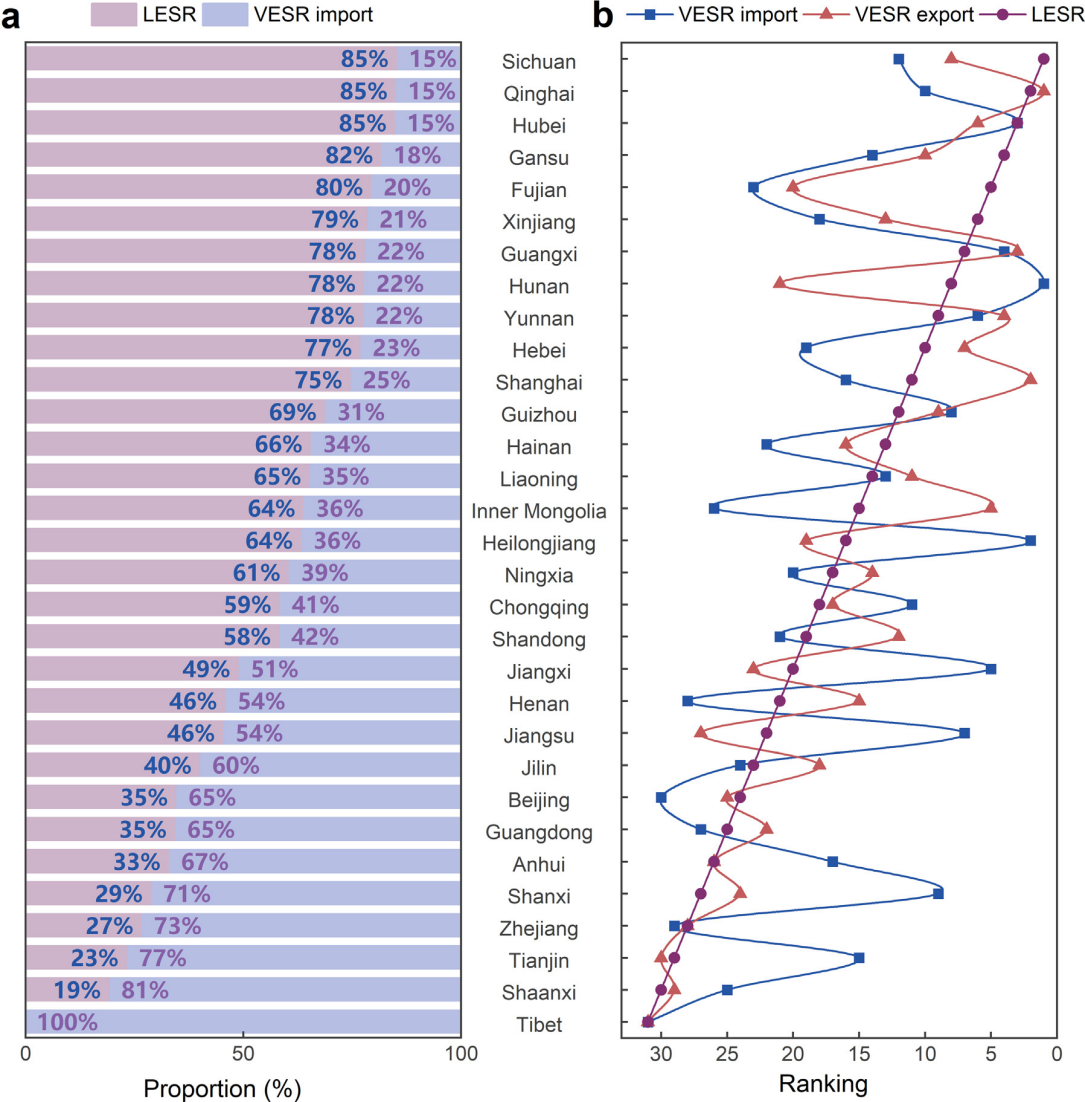
**Comparison of LESR, VESR exports, and VESR imports across regions in China in 2017.** (a) The proportion of VESR imports and LESR by region. (b) Rankings of LESR, VESR exports, and VESR imports by region.

VESR imports contributed over 50% of ESR in 12 out of 31 regions in 2017, including Tibet (100%), Shaanxi (81%), Tianjin (77%), Zhejiang (73%), and Shanxi (71%) (Fig. 4a). Notably, Tibet’s ESR was entirely trade-driven (i.e., from VESR imports), indicating its vulnerability to external energy shortages despite lacking LESR. Fig. 4b reveals similar rankings of LESR and VESR exports across regions, suggesting that regions with high local energy shortages often serve as major VESR exporters.

**Fig. 5:**
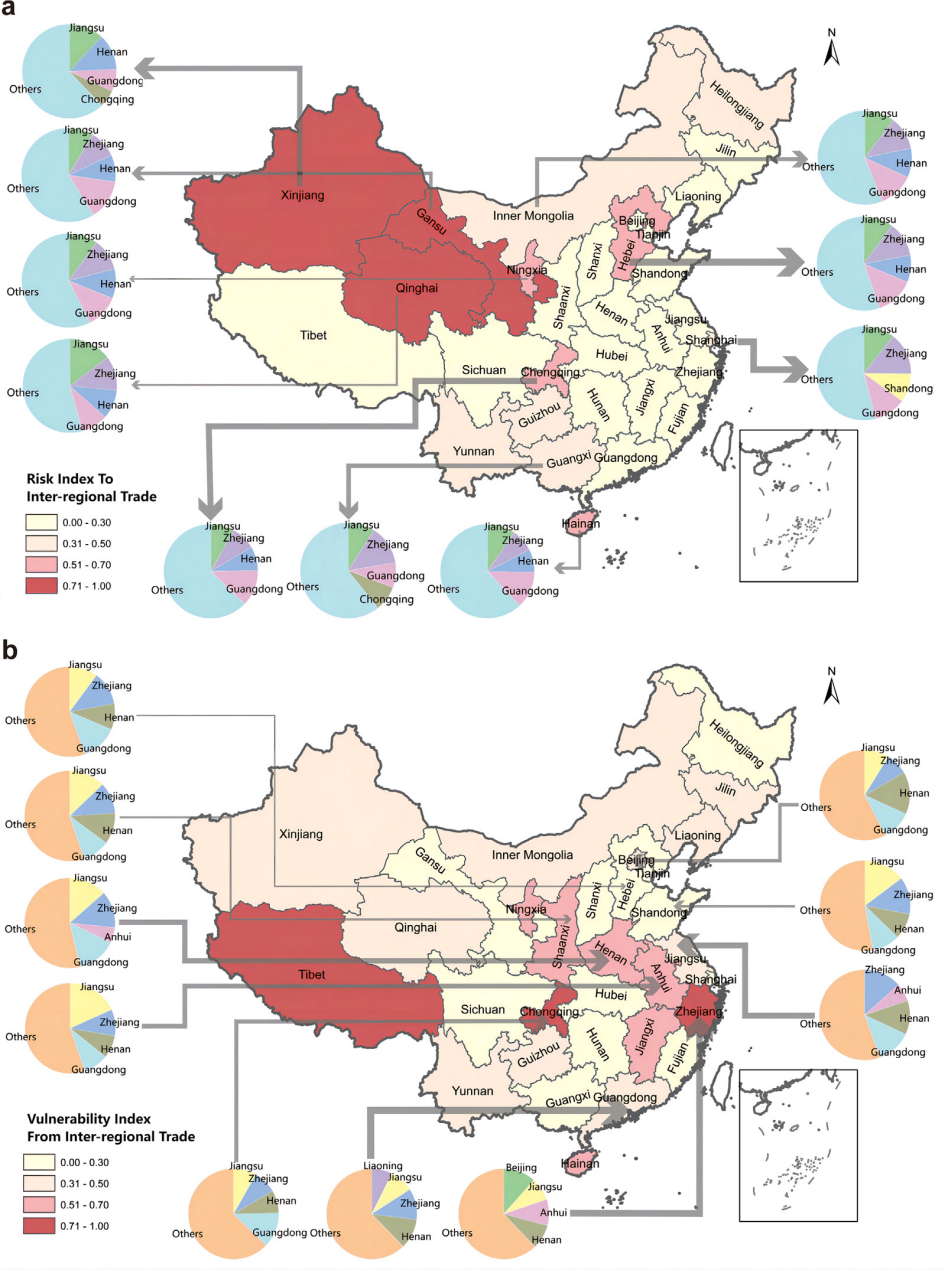
**The VESR exports (a) and imports (b) of major regions in China in 2017.** Note: The arrows flow from exporting to importing regions, with arrow widths proportional to VESR trade values. Risk indices (a) represent VESR exports normalized by regional total output, while Vulnerability indices (b) represent VESR imports normalized by regional total output. Map approval number: GS(2019)1822.

Fig. 5a demonstrates that the VESR within China’s trade network predominantly stems from Hebei (183.3 billion RMB), Shanghai (138.1 billion RMB), Chongqing (95.4 billion RMB), Xinjiang (87.7 billion RMB), and Guangxi (75.2 billion RMB). These regions exert considerable influence on other regions through their VESR exports. As the economic size of a region expands, the impacts of energy shortage would increase proportionally. To isolate the effect of economic size and better assess the inherent risk of VESR propagation, we introduce the concept of the VESR export per unit of output, defined as the RI. A higher RI indicates that a region poses a greater risk to external areas per unit of economic output due to local energy shortages. Regions with the highest RI values, including Qinghai, Xinjiang, Gansu, Hainan, and Ningxia, represent the most critical risk nodes within China’s trade network.

Fig. 5b maps the VESR imports across major regions and identifies their primary sources. The regions with the highest VESR imports include Guangdong (205.8 billion RMB), Zhejiang (172.1 billion RMB), Jiangsu (162.1 billion RMB), Henan (128.5 billion RMB), and Anhui (84.3 billion RMB). Although these regions do not face significant LESR directly, they are highly sensitive to energy shortages in upstream regions due to their reliance on imported goods. To account for differences in regional economic scales, we normalize VESR imports by total economic output to define the VI. Regions with higher values are highly vulnerable to VESR imports. We find that Tibet, Chongqing, Zhejiang, Shaanxi, Hainan, Ningxia, Anhui, Henan, and Jiangxi have the highest VI values, making them more susceptible to the effects of VESR imports. While Tibet’s VESR imports are relatively low, its high VI stems from its small economic output, which amplifies the relative impact of VESR imports.

#### 4.3.2 Region-sector level

**Fig. 6:**
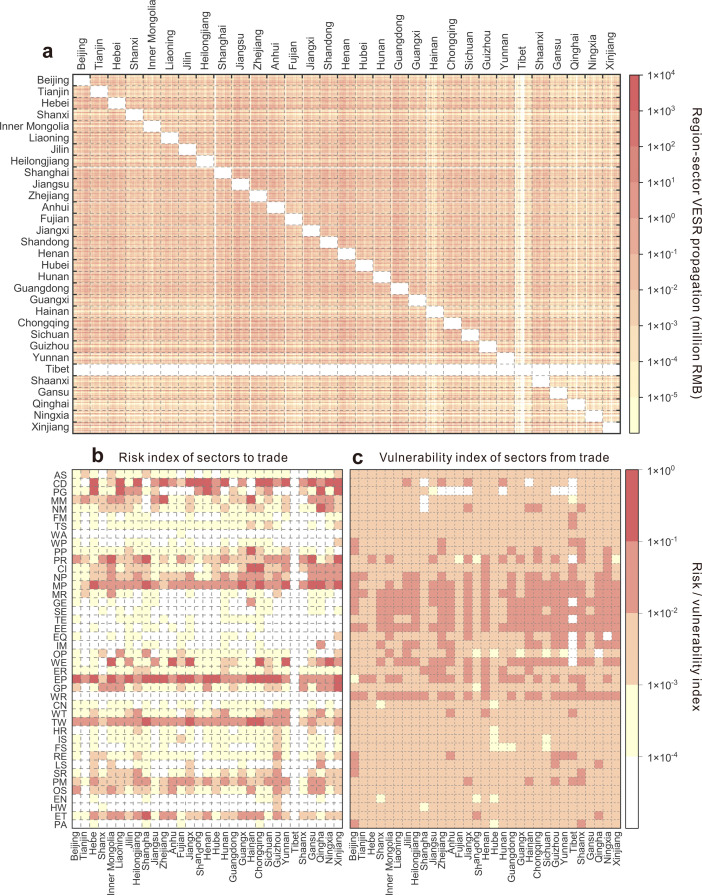
**VESR Propagation (a), RI values (b), and VI values (c) among region-sectors across China in 2017.** Note: In Panel (a), the rows and columns represent the origin and destination of VESR propagation, respectively, with each point in the heatmap indicating the VESR propagation from the origin region-sector (row) to the destination region-sector (column).

Fig. 6a illustrates the propagation of VESR across China at the region-sector level in 2017. VESR exports are primarily concentrated in energy-intensive sectors in regions with limited energy resources, particularly the “Metal Smelting and Pressing” sectors in Hebei (156.9 billion RMB), Henan (71.9 billion RMB), Shanghai (70.6 billion RMB), Jiangsu (59.8 billion RMB), and Guangxi (53.6 billion RMB) (Table S3). These energy-intensive upstream sectors served as key sources of VESR propagation within the national trade network.

In contrast, the major VESR importers include the “Water Production and Supply” sectors in Zhejiang (34.4 billion RMB) and Chongqing (26.9 billion RMB), as well as the “Metal Smelting and Pressing,” “Metal Products” and “Electrical equipment” sectors in Guangdong, with VESR imports of 26.4 billion RMB, 25.5 billion RMB, and 23.5 billion RMB, respectively (Table S4). These mainstream sectors are tightly coupled with upstream energy-intensive sectors, resulting in significant supply chain dependencies. Energy shortages in the upstream region-sectors directly constrain raw material supplies to downstream region-sectors, reducing their economic output. Among the 1302 region-sectors analyzed, 942 exhibited VESR imports exceeding 50% of their ESR (Fig. S3). For instance, the “Agriculture Services” and “Coal Mining and Dressing” sectors in Beijing derive most of their risk exposure from trade networks rather than local shortages. In Tibet, all sectors derived their ESR entirely from VESR imports.

To account for differences in economic scale, VESR export and import values were standardized for each region-sector, yielding the RI (Fig. 6b) and VI (Fig. 6c) metrics. Tables S5 and S6 list the top 100 region-sectors with the highest RI and VI. The region-sectors with the highest RI include the “Metal Ore Mining” sector in Hainan, the “Petroleum and Natural Gas” sector in Qinghai, the “Metal Smelting and Pressing” sector in Shanghai, and the “Electricity and Heat Production” sectors in Beijing and Chongqing. These sectors demonstrate exceptionally high VESR exports per unit of output, serving as critical risk sources within the trade network. The region-sectors with the highest VI comprise the “Metal Products” sector in Hainan, the “Metal Smelting and Pressing” and “Electricity and Heat Production” sectors in Tibet, the “Waste” sector in Chongqing, and the “Electricity and Heat Production” sector in Guangdong. These sectors exhibit elevated VESR imports per unit of output, representing significant vulnerable nodes in the trade network.

We further identified critical pairs in terms of VESR propagation. The “Metal Smelting and Pressing” sector in Hebei to the “Water Production and Supply” sector in Zhejiang formed the most tightly connected region-sector pair in VESR propagation, where energy shortage in Hebei’s “Metal Smelting and Pressing” sector led to potential economic losses of 4.7 billion RMB in the downstream “Water Production and Supply” sector in Zhejiang. The “Metal Smelting and Pressing” sector in Shanghai to the “Water Production and Supply” sector in Zhejiang was the second-most-important pair. Energy shortage in Shanghai’s “Metal Smelting and Pressing” sector resulted in potential economic losses of 4.2 billion RMB in the downstream “Water Production and Supply” sector in Zhejiang. Other significant pairs in terms of VESR propagation included the “Metal Smelting and Pressing” sector in Hebei to the “Metal Smelting and Pressing” sector in Guangdong (3.9 billion RMB), the “Metal Smelting and Pressing” sector in Hebei to the “Metal Products” sector in Guangdong (3.8 billion RMB), and the “Metal Smelting and Pressing” sector in Hebei to the “Water Production and Supply” sector in Chongqing (3.8 billion RMB).

The propagation of VESR at the region-sector level can be categorized into two distinct types based on sectoral characteristics. For sectors categorized as producers of essential raw materials, such as “Metal Smelting and Pressing,” the impact of production disruptions can be partially mitigated if downstream sectors can find substitutes with acceptable price differences and transportation costs. However, for technology-intensive sectors such as the “Electronic Equipment” sector, substitution is often infeasible due to strict technical specifications and specialized design requirements [47]. Consequently, energy shortages that constrain production in these sectors can trigger substantial indirect losses across dependent industries that require intermediate inputs from these sectors.

Despite the overall reduction in China’s VESR, certain region-sectors experienced notable increases in VESR imports and exports. VESR exports increased in 513 region-sectors during this period, particularly in the “Metal Smelting and Pressing” sectors of Shanghai (51.3 billion RMB), Jiangsu (48.1 billion RMB), and Inner Mongolia (23.9 billion RMB), among others (see SI Table S7). Meanwhile, VESR imports grew in 390 region-sectors, notably in the “Water Production and Supply” sectors of Zhejiang (34.3 billion RMB), Chongqing (26.9 billion RMB), and Shaanxi (17.0 billion RMB), among others (see SI Table S8). These region-sectors with substantial growth in VESR imports or exports warrant particular attention from policymakers.

### 4.4 Measuring the concurrence of VESR

In addition to VESR propagation analysis, we quantified the concentration of VESR imports across region-sectors to assess their vulnerability to external energy shortages. The concentration of VESR imports is inversely related to regional-sectoral resilience. When region-sectors depend on limited upstream VESR suppliers, the probability of concurrent energy shortages among these suppliers increases, elevating their exposure to external energy supply risk. To measure this concentration (VESR import concentration), we employed the Herfindahl-Hirschman Index (HHI).

**Fig. 7:**
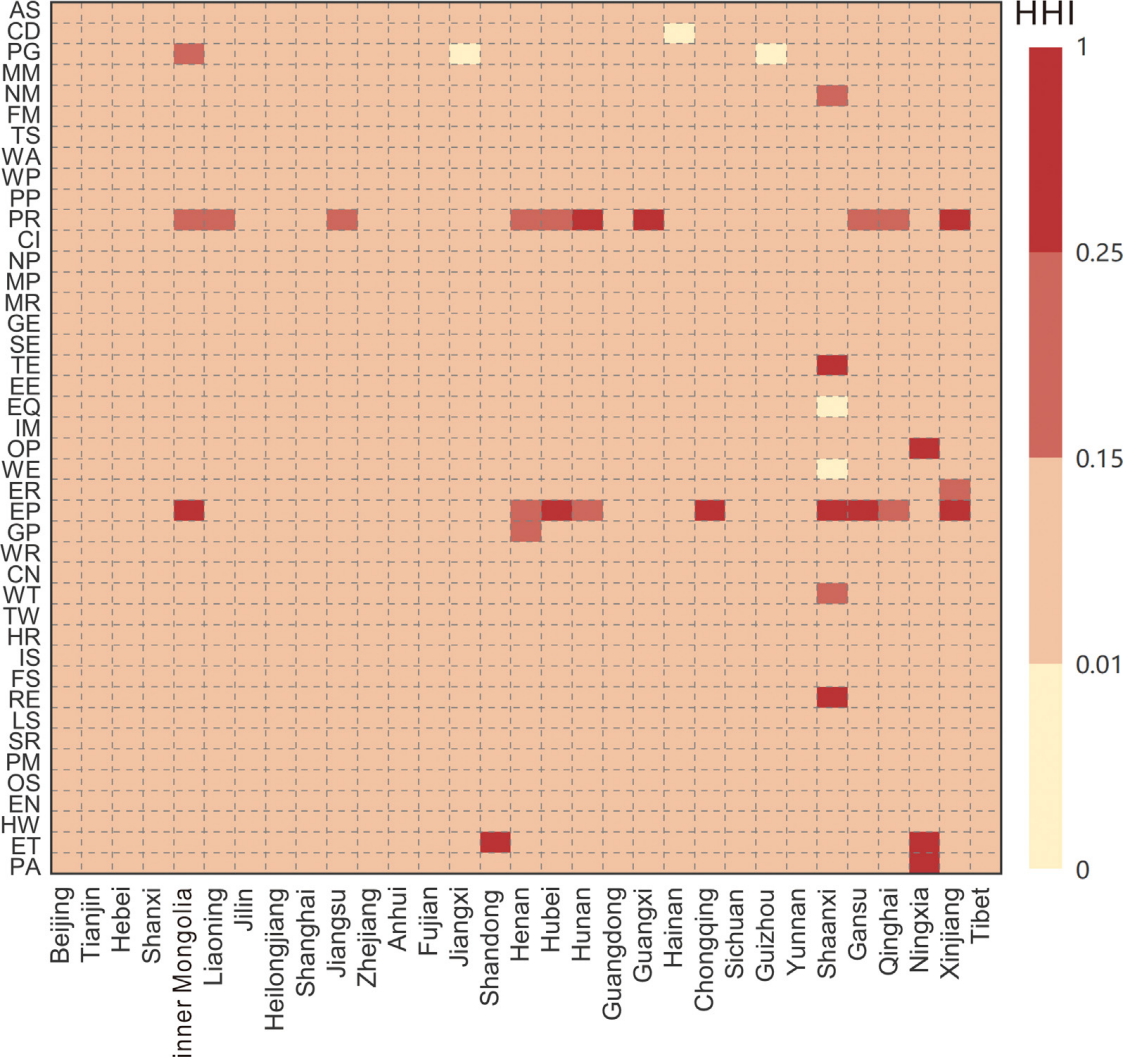
**The Herfindahl index for each region-sector in China in 2017**.

14 region-sectors exhibited high VESR import concentration (HHI>0.25), such as the “Transport equipment” and “Electricity and Heat Production” sectors in Tibet, the “Entertainment” sector in Shandong, the “Electricity and Heat Production” and “Petroleum Refining” sectors in Ningxia (Fig. 7). These region-sectors’ VESR imports are dominated by few suppliers, increasing their vulnerability to external energy shortages. Additionally, 14 region-sectors showed moderate VESR import concentration (0.15≤HHI≤0.25), such as the “Real Estate” sector in Tibet, the “Electricity and Heat Production” sector in Gansu, and the “Petroleum Refining” sectors in Hebei and Inner Mongolia, and Gansu, where limited upstream suppliers make them susceptible to external shocks. Notably, several region-sectors, including the “Gas Production” and “Electrical equipment” sectors in Tibet, and the “Waste” sector in Qinghai, require particular attention from policymakers despite their relatively low VESR import values due to moderate concentration.

Temporal analysis of HHI further revealed substantial structural change in VESR import concentration between 2012 and 2015 (Fig. S4). The number of region-sectors with highly concentrated VESR imports increased from 3 in 2012 to 14 in 2017, while those with moderate concentration rose from 0 to 14. Although the total VESR showed a declining trend during 2012–2017, the increased concentration of VESR imports has weakened the trade network’s resilience against energy shortages.

## 5 Discussion

### 5.1 Policy implications

Energy is the foundation of economic production activities. When energy shortages occur in a region, they not only disrupt local production but also propagate through interregional supply chains, potentially causing indirect economic losses in other regions. Although China has implemented energy management strategies focusing on improving energy efficiency and adjusting energy structure, these approaches often localize energy shortage issues [[48], [49]], overlooking the systemic risk that local energy shortages pose to the trade network. By examining the VESR propagation mechanisms across region-sectors during 2012–2017, this study provides new insights for enhancing the resilience of China’s trade network to energy shortages. This research has significant implications for China’s ongoing energy transition. As China accelerates its transition from fossil fuels to renewable energy [50,51], potential supply gaps arising from temporal mismatches between renewable deployment and fossil fuel capacity reduction have led to frequent regional power outages and restrictions [52]. Within this context, understanding and managing VESR propagation becomes increasingly urgent.

Primary VESR exporters in China’s trade network include Hebei, Shanghai, Chongqing, Xinjiang, and Guangxi. These regions emerge as key VESR sources due to their domestic energy shortages and dominant role in bulk commodity exports. At the region-sector level, the “Metal Smelting and Pressing” sector in Hebei, Henan, Shanghai, Jiangsu, and Guangxi dominates VESR exports. These region-sectors are energy-intensive and provide substantial input supplies to downstream producers, resulting in large-scale VESR exports. To reduce VESR exports and ensure energy supply and demand balance in these regions and sectors, strategic interventions such as (1) enhancing energy utilization efficiency through technology adoption incentives, and prioritizing advanced energy-saving technologies; (2) diversifying energy supply portfolios through renewable infrastructure development, and improving interregional energy transmission to enhance resilience to energy shortages.

Major VESR importers in China’s trade network are Guangdong, Zhejiang, Jiangsu, Henan, and Anhui. These regions have become major destinations for VESR propagation due to their reliance on imports from regions affected by energy shortages. At the region-sector level, the “Water Production and Supply” sectors in Zhejiang and Chongqing, as well as the “Metal Smelting and Pressing,” “Metal Products,” and “Electrical Equipment” sectors in Guangdong, dominate VESR imports. The reliance of these region-sectors on inputs from upstream sectors experiencing energy shortages heightens the risk of supply chain disruptions, leaving them highly susceptible to external energy shortages. To reduce VESR imports, policymakers should adopt targeted measures to decrease the reliance of these regions and sectors on imports from suppliers facing energy shortages. Recommended strategic interventions include (1) diversifying upstream input supply by sourcing from multiple suppliers can reduce reliance on a single source; (2) promoting partnerships with suppliers that have sufficient energy supplies can help prevent supply chain disruptions caused by energy shortages [53].

We also identified key pairs of VESR propagation, including the “Metal Smelting and Pressing” sector in Hebei to the “Water Production and Supply” sector in Zhejiang, the “Metal Smelting and Pressing” sector in Hebei to the “Metal Smelting and Pressing” sector in Guangdong, and the “Metal Smelting and Pressing” sector in Hebei to its counterpart sector in Guangdong. These region-sector pairs demonstrate significant VESR linkages, where enhanced collaboration is essential for VESR mitigation. Potential collaborative measures include (1) developing cross-regional early warning mechanisms to facilitate timely communication of upstream energy shortages and optimize downstream supply chains; (2) establishing inter-regional technology cooperation to promote the transfer and implementation of energy efficiency technologies.

### 5.2 Sensitivity analysis

**Fig. 8:**
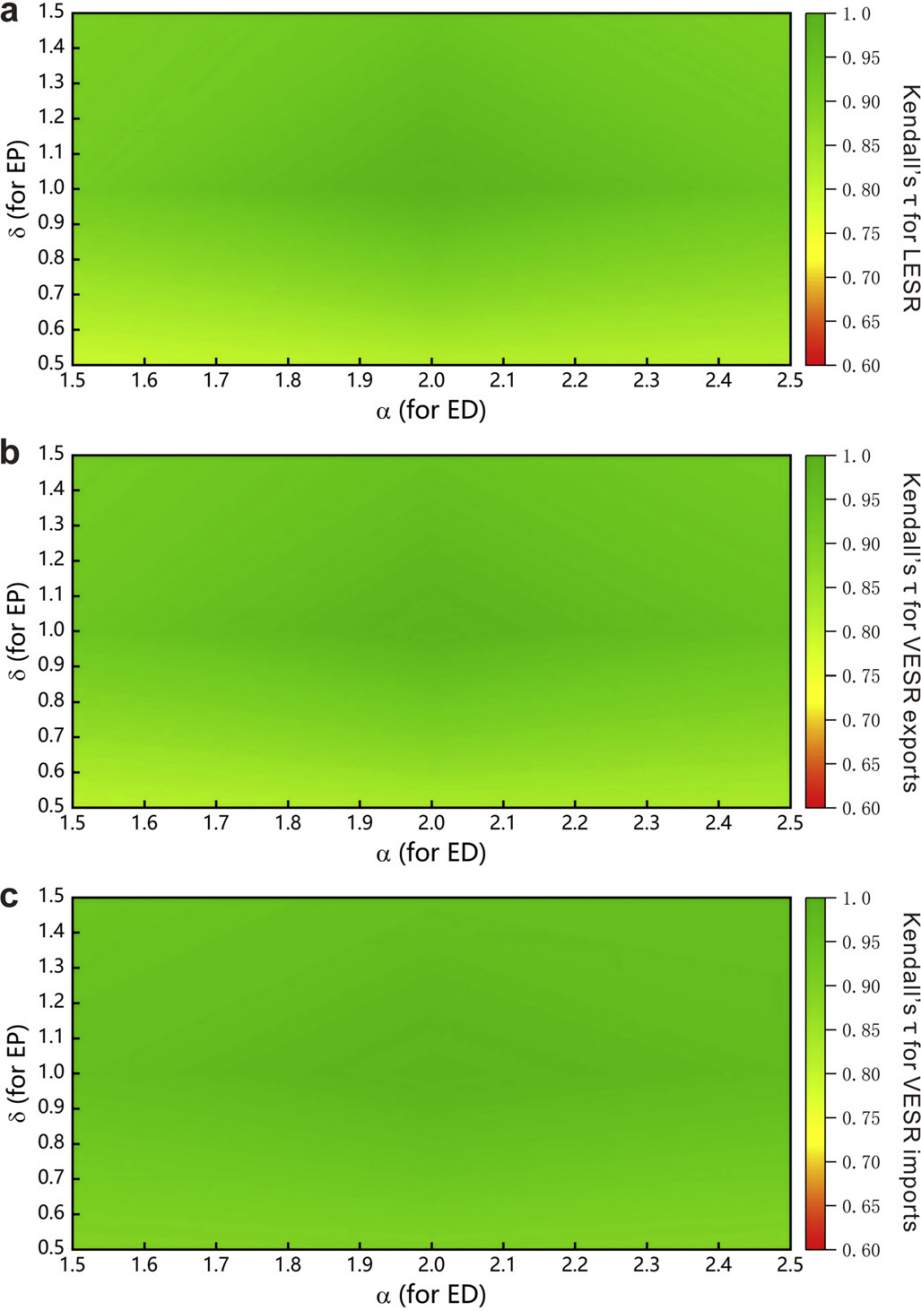
**Kendall correlation coefficients of rankings using different parameter pairs with the baseline scenario (where σ = 1 and α = 2).** (a) LESR, (b) VESR exports, and (c) VESR imports. Green areas indicate high correlations (indicating robust results), while yellow and red areas represent moderate correlations.

The EP and ED results are primarily determined by parameter selection. Parameter σ governs the heterogeneity of EP across regions (Eq. 3), while parameter α defines the threshold for ED (Eq. 7). To assess the robustness of the results, we performed calculations for 9 pairs of (σ, α). Kendall correlation coefficient was computed by comparing all (σ, α) pairs with the baseline scenario (where σ = 1 and α = 2). Fig. 8a–c presents the Kendall correlation coefficients for the rankings of LESR, VESR exports, and VESR imports, respectively. Coefficients approaching 1 (indicated by the green) indicate minimal divergence in rankings from the baseline scenario [54], reflecting the robustness of the results. Analysis reveals high robustness for LESR and VESR exports and exceptional robustness for VESR imports. While LESR and VESR exports rely on single-point measurements, VESR imports originate from multiple sectors, where the national trade system’s aggregation effect mitigates potential estimation biases.

### 5.3 Limitations and future perspectives

This study has certain limitations. First, estimating energy consumption for service sectors at the regional level may introduce some uncertainty, which could be addressed with more detailed sector-level data. Second, this study did not account for the availability of homogeneous substitutes for upstream inputs during supply chain disruptions, which limits the comprehensiveness of its resilience evaluation. Subsequent studies may refine the risk assessment framework by incorporating scenario analysis and game theory to simulate the supply strategies and market dynamics of substitutes. Third, the research primarily focuses on the regional and sectoral levels, with inadequate integration of firm-level data. As firms adopt unique strategies in addressing supply chain risks, integrating firm-specific data (e.g., inventory management practices and supplier diversification) would enable a deeper understanding of such risks. Future work could develop a multi-level analytical framework (firm-region-nation) and combine micro-level firm behaviors with macroeconomic data to clarify the transmission mechanisms of supply chain risks.

## 6 Conclusion

This study quantified the potential direct (LESR) and indirect (VESR) economic losses stemming from local energy shortage. In 2017, China’s VESR accounted for 41% of the ESR, highlighting that a substantial portion of the economic impacts of energy shortages occur beyond geographical borders. We further identified the hotspots of VESR propagation within the trade network. VESR exporters are primarily concentrated in energy-intensive sectors within energy-scarce regions (e.g., the “Metal Smelting and Pressing” sector in Hebei), while VESR importers are widely distributed, including sectors in regions without energy shortages (e.g., all sectors in Tibet). This work provides a comprehensive assessment framework for assessing the potential indirect economic loss caused by energy shortages, addressing the gaps in previous research on VESR. The findings offer policymakers key hotspots for prioritizing VESR mitigation and emphasize the importance of reducing VESR to strengthen the resilience of the trade network. The methodological framework employed in this study can be extended to global-scale analysis to address the growing international energy supply challenges.

## CRediT authorship contribution statement

**Hanlei Wang:** Writing – original draft, Visualization, Software, Methodology, Data curation. **Hui Li:** Writing – review & editing, Funding acquisition, Conceptualization. **Yulei Xie:** Supervision, Conceptualization. **Frederick Kwame Yeboah:** Writing – review & editing. **Zhiyao Ding:** Data curation. **Gengyuan Liu:** Supervision. **Yixuan Wang:** Conceptualization.

## Declaration of competing interest

The authors declare that they have no conflicts of interest in this work.

## Appendix Supplementary materials

Image, application 1
