# Supplementary material for: Virtual energy shortage risk to trade network in China
Source: Fundam Res. 2025 Nov 13;6(2):647–58. doi: 10.1016/j.fmre.2025.11.005 (PMC13069848; doi:10.1016/j.fmre.2025.11.005)
Supplement: Supplementary file 1 [file mmc1.docx]

**Supplementary Information**

for

**Virtual energy shortage risk to trade network in China**

Pages: 19

Figures: 4

Tables: 8

The Supplementary Information provides supplemental Figures and Tables.


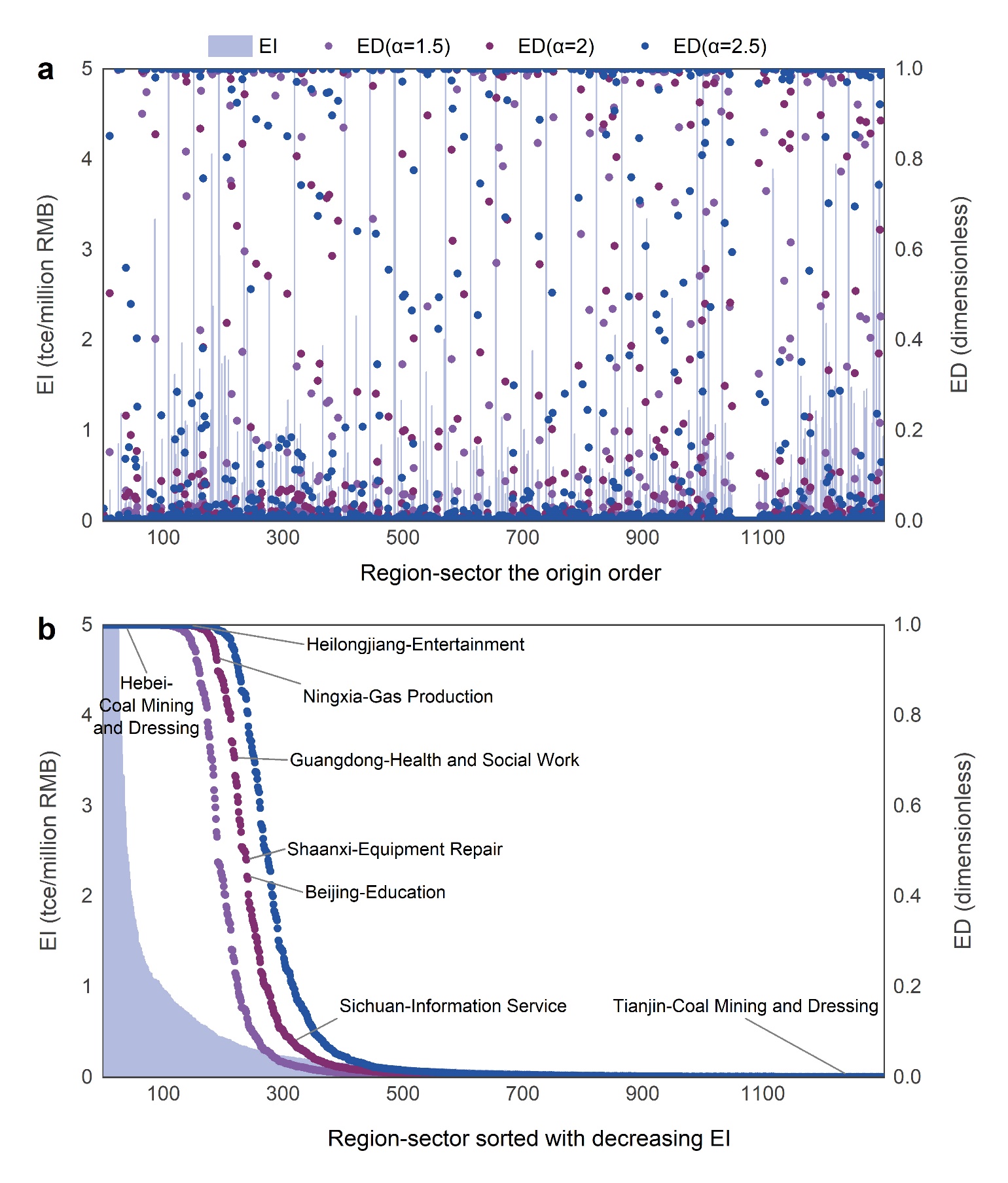


**Figure S1.** The EI and corresponding ED of China's region-sectors. Note: Panel (a) displays the data in the original order of the underlying MRIO dataset, while Panel (b) rearranges the data in descending order of EI. The dataset includes 31 regions and 42 sectors, resulting in a total of 31 × 42 = 1302 region-sector combinations.


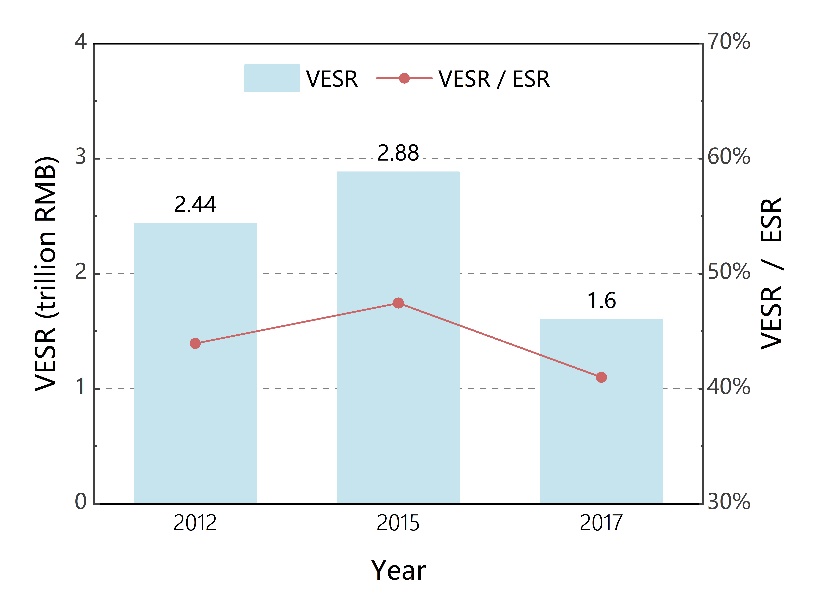


**Figure S2.** Temporal evolution of VESR and its share in ESR from 2012 to 2017.


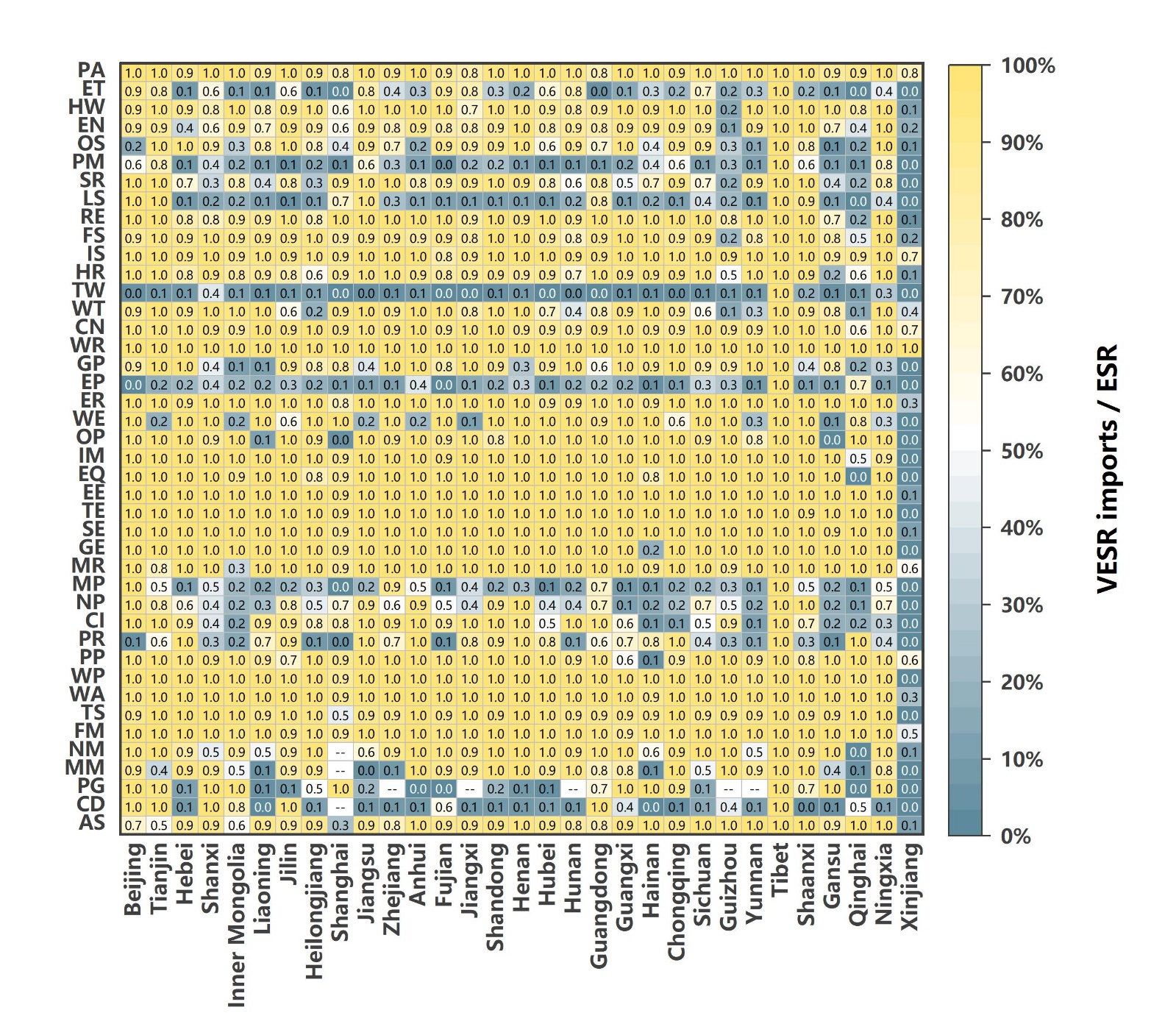


**Figure S3.** The share of VESR imports in ESR by region-sector. Note: Columns represent different sectors (full names of the sectors are provided in Table S2).


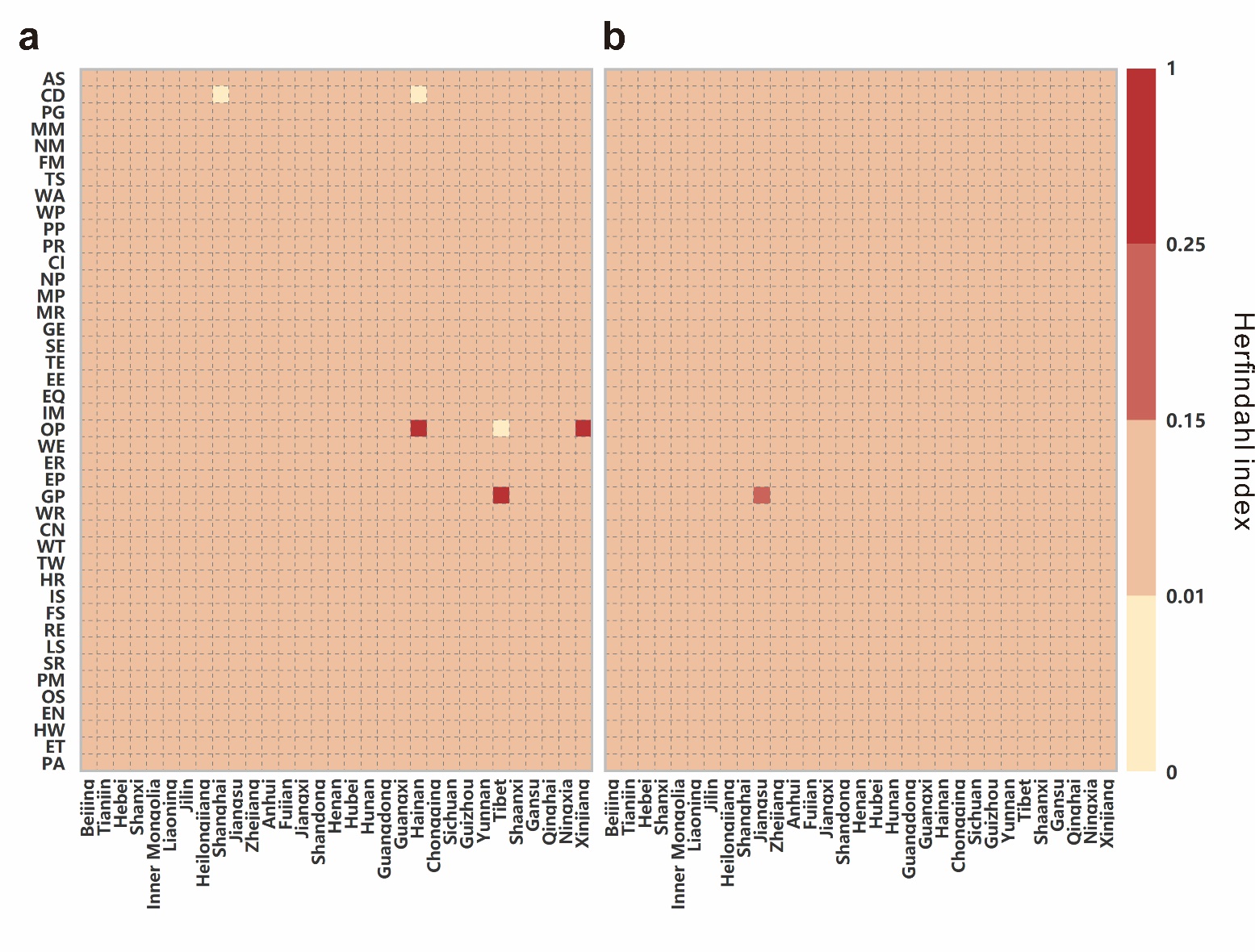


**Figure S4.** The Herfindahl index for each region-sector in China in 2012 (a) and 2015 (b). Note: Columns represent different sectors (full names of the sectors are provided in Table S2).

# Table S1. Conversion Factors from Physical Units to Coal Equivalent

| **Energy** | **Conversion Factor** | **Energy** | **Conversion Factor** |
| --- | --- | --- | --- |
| Coal | 0.7143 kgce/kg | Coke | 0.9714 kgce/kg |
| Crude Oil | 1.4286 kgce/kg | Gasoline | 1.4714 kgce/kg |
| Kerosene | 1.4714 kgce/kg | Diesel | 1.4571 kgce/kg |
| Fuel Oil | 1.4286 kgce/kg | Liquefied Petroleum Gas | 1.7143 kgce/kg |
| Natural Gas | 1.1000~1.3300 kgce/cu.m | Electricity | 0.1229 kgce/kWh |

# Table S2. Sector classifications and their abbreviations

| **Sector** | **Abbreviation** | **Sector** | **Abbreviation** |
| --- | --- | --- | --- |
| Agriculture Services | AS | Other Manufacturing Products | OP |
| Coal Mining and Dressing | CD | Waste | WE |
| Petroleum and Natural Gas | PG | Equipment Repair | ER |
| Metal Ore Mining | MM | Electricity and Heat Production | EP |
| Nonmetal and Other Ores Mining | NM | Gas Production | GP |
| Food Manufacturing | FM | Water Production and Supply | WR |
| Textiles | TS | Construction | CN |
| Wearing Apparel | WA | Wholesale and Retail Trade | WT |
| Wood Products | WP | Transportation and Warehousing | TW |
| Paper and Printing | PP | Hotels and Restaurants | HR |
| Petroleum Refining | PR | Information Service | IS |
| Chemical Industry | CI | Financial Service | FS |
| Nonmetal Mineral Products | NP | Real Estate | RE |
| Metal Smelting and Pressing | MP | Leasehold and Business Services | LS |
| Metal Products | MR | Scientific Research | SR |
| General Equipment | GE | Public Facility Management | PM |
| Special Equipment | SE | Other Services | OS |
| Transport Equipment | TE | Education | EN |
| Electrical Equipment | EE | Health and Social Work | HW |
| Electronic Equipment | EQ | Entertainment | ET |
| Instruments and Meters | IM | Public Administration | PA |

# Table S3. Top 100 region-sectors with the highest VESR export in 2017

| **Rank** | **region-sector** | **VESR export**  **(billion RMB)** | **Rank** | **region-sector** | **VESR export**  **(billion RMB)** |
| --- | --- | --- | --- | --- | --- |
| 1 | Hebei-MP | 156.85 | 51 | Sichuan-EP | 7.25 |
| 2 | Henan-MP | 71.92 | 52 | Xinjiang-CI | 7.11 |
| 3 | Shanghai-MP | 70.57 | 53 | Heilongjiang-TW | 7.02 |
| 4 | Jiangsu-MP | 59.76 | 54 | Guizhou-CD | 7.01 |
| 5 | Guangxi-MP | 53.55 | 55 | Jilin-MP | 6.85 |
| 6 | Shanghai-TW | 38.42 | 56 | Shanghai-ET | 6.77 |
| 7 | Xinjiang-PG | 35.59 | 57 | Heilongjiang-PR | 6.73 |
| 8 | Liaoning-MP | 34.61 | 58 | Hebei-CD | 6.44 |
| 9 | Chongqing-CI | 33.80 | 59 | Ningxia-CI | 6.24 |
| 10 | Hunan-MP | 29.89 | 60 | Hunan-NP | 6.21 |
| 11 | Yunnan-MP | 28.99 | 61 | Hunan-TW | 6.19 |
| 12 | Fujian-MP | 28.10 | 62 | Inner Mongolia-PR | 6.04 |
| 13 | Henan-CD | 26.92 | 63 | Hainan-TW | 5.98 |
| 14 | Zhejiang-TW | 25.65 | 64 | Sichuan-PG | 5.88 |
| 15 | Gansu-MP | 25.54 | 65 | Heilongjiang-CD | 5.83 |
| 16 | Shandong-CD | 24.83 | 66 | Guangdong-TW | 5.69 |
| 17 | Inner Mongolia-MP | 24.43 | 67 | Shandong-PG | 5.68 |
| 18 | Shandong-MP | 24.35 | 68 | Guangxi-TW | 5.65 |
| 19 | Chongqing-MP | 22.32 | 69 | Hebei-TW | 5.50 |
| 20 | Sichuan-TW | 21.93 | 70 | Xinjiang-CD | 5.35 |
| 21 | Chongqing-TW | 21.28 | 71 | Hunan-PR | 5.25 |
| 22 | Xinjiang-MP | 20.19 | 72 | Guangdong-EP | 5.20 |
| 23 | Hubei-MP | 18.42 | 73 | Jiangsu-EP | 5.17 |
| 24 | Jiangxi-MP | 16.94 | 74 | Shandong-TW | 5.12 |
| 25 | Henan-TW | 16.38 | 75 | Jiangxi-NP | 5.03 |
| 26 | Guizhou-MP | 16.13 | 76 | Shaanxi-MP | 4.92 |
| 27 | Xinjiang-PR | 14.92 | 77 | Ningxia-MP | 4.68 |
| 28 | Shanghai-PR | 12.85 | 78 | Ningxia-CD | 4.50 |
| 29 | Guangxi-NP | 12.83 | 79 | Shaanxi-PR | 4.43 |
| 30 | Hainan-CI | 12.64 | 80 | Liaoning-PG | 4.42 |
| 31 | Inner Mongolia-CI | 12.15 | 81 | Gansu-CI | 4.41 |
| 32 | Sichuan-CD | 12.03 | 82 | Fujian-TW | 4.24 |
| 33 | Guizhou-WT | 11.54 | 83 | Yunnan-CI | 4.18 |
| 34 | Yunnan-CD | 11.16 | 84 | Sichuan-CI | 4.09 |
| 35 | Qinghai-PG | 10.66 | 85 | Chongqing-CD | 4.00 |
| 36 | Shaanxi-CD | 10.43 | 86 | Tianjin-MP | 3.88 |
| 37 | Heilongjiang-WT | 10.38 | 87 | Sichuan-PR | 3.83 |
| 38 | Hubei-TW | 10.15 | 88 | Jiangxi-EP | 3.69 |
| 39 | Jiangxi-TW | 9.96 | 89 | Fujian-PR | 3.61 |
| 40 | Beijing-EP | 9.25 | 90 | Shanxi-MP | 3.57 |
| 41 | Gansu-PR | 8.53 | 91 | Zhejiang-EP | 3.46 |
| 42 | Jilin-TW | 8.50 | 92 | Chongqing-NP | 3.42 |
| 43 | Jiangsu-TW | 8.28 | 93 | Anhui-TW | 3.39 |
| 44 | Chongqing-EP | 8.28 | 94 | Liaoning-CD | 3.33 |
| 45 | Anhui-CD | 7.99 | 95 | Henan-EP | 3.14 |
| 46 | Beijing-TW | 7.85 | 96 | Shanghai-EP | 3.06 |
| 47 | Sichuan-MP | 7.80 | 97 | Fujian-EP | 2.99 |
| 48 | Anhui-MP | 7.62 | 98 | Hunan-CD | 2.96 |
| 49 | Qinghai-MP | 7.59 | 99 | Hebei-EP | 2.91 |
| 50 | Guangdong-MP | 7.28 | 100 | Liaoning-PR | 2.88 |

**Note:** Letters following regions denote sectors (see Table S2 for the full names of sectors).

# Table S4. Top 100 region-sectors with the highest VESR import in 2017

| **Rank** | **region-sector** | **VESR import**  **(billion RMB)** | **Rank** | **region-sector** | **VESR import**  **(billion RMB)** |
| --- | --- | --- | --- | --- | --- |
| 1 | Zhejiang-WR | 34.38 | 51 | Hebei-WR | 6.82 |
| 2 | Chongqing-WR | 26.89 | 52 | Henan-EE | 6.68 |
| 3 | Guangdong-MP | 26.38 | 53 | Zhejiang-ER | 6.68 |
| 4 | Guangdong-MR | 25.45 | 54 | Shanghai-WR | 6.61 |
| 5 | Guangdong-EE | 23.45 | 55 | Henan-SE | 6.51 |
| 6 | Jiangsu-MP | 19.11 | 56 | Guangdong-SE | 6.31 |
| 7 | Guangdong-EQ | 18.84 | 57 | Shanxi-WR | 6.29 |
| 8 | Jiangsu-EE | 18.75 | 58 | Shandong-TE | 6.17 |
| 9 | Shaanxi-WR | 16.98 | 59 | Henan-FM | 6.15 |
| 10 | Jiangsu-WR | 16.94 | 60 | Guangxi-WR | 6.14 |
| 11 | Zhejiang-MP | 16.38 | 61 | Henan-GE | 6.09 |
| 12 | Yunnan-WR | 16.10 | 62 | Guangdong-NP | 6.06 |
| 13 | Zhejiang-EE | 15.88 | 63 | Hainan-WR | 5.99 |
| 14 | Jiangsu-CI | 15.02 | 64 | Jiangsu-SE | 5.88 |
| 15 | Guangdong-WR | 14.75 | 65 | Anhui-CI | 5.62 |
| 16 | Zhejiang-GE | 14.58 | 66 | Chongqing-MP | 5.60 |
| 17 | Henan-MP | 14.13 | 67 | Zhejiang-TE | 5.56 |
| 18 | Chongqing-TE | 12.37 | 68 | Zhejiang-SE | 5.53 |
| 19 | Guangdong-TE | 11.99 | 69 | Zhejiang-PP | 5.27 |
| 20 | Henan-WR | 11.98 | 70 | Zhejiang-EQ | 5.23 |
| 21 | Zhejiang-CI | 11.78 | 71 | Heilongjiang-WR | 5.15 |
| 22 | Beijing-WR | 11.36 | 72 | Henan-TE | 5.14 |
| 23 | Liaoning-WR | 11.27 | 73 | Tianjin-WR | 5.09 |
| 24 | Henan-NP | 11.15 | 74 | Zhejiang-TS | 4.92 |
| 25 | Guangdong-CI | 10.84 | 75 | Jiangsu-ER | 4.92 |
| 26 | Zhejiang-MR | 10.79 | 76 | Shandong-WR | 4.91 |
| 27 | Jiangxi-WR | 10.34 | 77 | Beijing-HR | 4.85 |
| 28 | Guizhou-WR | 10.28 | 78 | Jiangsu-IS | 4.85 |
| 29 | Anhui-MP | 9.98 | 79 | Anhui-MR | 4.77 |
| 30 | Sichuan-WR | 9.73 | 80 | Anhui-WT | 4.71 |
| 31 | Jiangsu-EQ | 9.66 | 81 | Jiangsu-NP | 4.57 |
| 32 | Jiangsu-MR | 9.60 | 82 | Anhui-TE | 4.56 |
| 33 | Henan-CI | 9.55 | 83 | Guangdong-ER | 4.56 |
| 34 | Guangdong-GE | 9.38 | 84 | Jilin-TE | 4.49 |
| 35 | Anhui-WR | 9.35 | 85 | Guangdong-WA | 4.40 |
| 36 | Beijing-SR | 9.08 | 86 | Shandong-MP | 4.30 |
| 37 | Inner Mongolia-WR | 9.06 | 87 | Hebei-CI | 4.26 |
| 38 | Guangdong-PP | 8.74 | 88 | Jiangsu-RE | 4.18 |
| 39 | Hebei-MP | 8.58 | 89 | Hebei-MR | 4.10 |
| 40 | Jiangxi-MP | 8.40 | 90 | Shaanxi-TE | 4.03 |
| 41 | Anhui-EE | 8.38 | 91 | Shanghai-RE | 4.00 |
| 42 | Hunan-WR | 8.37 | 92 | Anhui-GE | 3.97 |
| 43 | Jiangsu-GE | 8.32 | 93 | Guangdong-RE | 3.97 |
| 44 | Henan-MR | 7.87 | 94 | Shaanxi-MP | 3.96 |
| 45 | Henan-WT | 7.78 | 95 | Beijing-ER | 3.94 |
| 46 | Beijing-RE | 7.65 | 96 | Liaoning-MP | 3.93 |
| 47 | Shandong-CI | 7.60 | 97 | Shanghai-WT | 3.83 |
| 48 | Jiangsu-TE | 7.51 | 98 | Tianjin-MP | 3.78 |
| 49 | Xinjiang-WR | 7.38 | 99 | Beijing-TE | 3.73 |
| 50 | Jilin-WR | 7.26 | 100 | Beijing-LS | 3.71 |

**Note:** Letters following regions denote sectors (see Table S2 for the full names of sectors).

# Table S5. Top 100 region-sectors with the highest RI in 2017

| **Rank** | **Region-sector** | **RI**  **(10^-2^)** | **Rank** | **Region-sector** | **RI**  **(10^-2^)** |
| --- | --- | --- | --- | --- | --- |
| 1 | Hainan-MM | 53.55 | 51 | Chongqing-WE | 11.59 |
| 2 | Qinghai-PG | 45.42 | 52 | Hainan-EP | 11.51 |
| 3 | Shanghai-MP | 44.20 | 53 | Liaoning-CD | 11.51 |
| 4 | Beijing-EP | 30.98 | 54 | Inner Mongolia-EP | 11.50 |
| 5 | Chongqing-EP | 30.97 | 55 | Zhejiang-CD | 11.33 |
| 6 | Shanghai-TW | 30.29 | 56 | Liaoning-PG | 10.75 |
| 7 | Yunnan-CD | 30.18 | 57 | Hebei-PG | 10.70 |
| 8 | Xinjiang-PG | 29.12 | 58 | Hubei-EP | 10.63 |
| 9 | Hainan-CI | 24.95 | 59 | Qinghai-NM | 10.62 |
| 10 | Sichuan-EP | 22.33 | 60 | Shanghai-EP | 10.51 |
| 11 | Jiangxi-EP | 22.23 | 61 | Qinghai-MP | 10.45 |
| 12 | Yunnan-WE | 21.52 | 62 | Shandong-CD | 10.25 |
| 13 | Chongqing-TW | 20.56 | 63 | Gansu-CD | 10.16 |
| 14 | Chongqing-CD | 19.25 | 64 | Hunan-CD | 10.12 |
| 15 | Shanghai-ET | 19.03 | 65 | Inner Mongolia-PR | 10.07 |
| 16 | Chongqing-MP | 18.38 | 66 | Guizhou-MP | 9.69 |
| 17 | Xinjiang-CD | 17.94 | 67 | Hunan-PR | 9.66 |
| 18 | Hainan-PP | 17.45 | 68 | Jilin-MP | 9.60 |
| 19 | Guangxi-MP | 16.80 | 69 | Inner Mongolia-MP | 9.45 |
| 20 | Sichuan-CD | 16.20 | 70 | Jilin-PG | 9.45 |
| 21 | Jiangxi-CD | 16.10 | 71 | Guangdong-EP | 9.24 |
| 22 | Chongqing-CI | 15.88 | 72 | Heilongjiang-PR | 8.92 |
| 23 | Henan-CD | 15.66 | 73 | Jilin-TW | 8.80 |
| 24 | Jiangxi-WE | 15.59 | 74 | Inner Mongolia-CI | 8.65 |
| 25 | Hebei-MP | 15.35 | 75 | Ningxia-EP | 8.62 |
| 26 | Gansu-MP | 15.01 | 76 | Liaoning-MP | 8.60 |
| 27 | Shanghai-PR | 14.89 | 77 | Hubei-PG | 8.58 |
| 28 | Ningxia-CD | 14.46 | 78 | Henan-MP | 8.58 |
| 29 | Heilongjiang-CD | 14.38 | 79 | Guangxi-NP | 8.52 |
| 30 | Yunnan-MP | 14.34 | 80 | Fujian-MP | 8.48 |
| 31 | Hainan-TW | 14.18 | 81 | Zhejiang-TW | 8.43 |
| 32 | Anhui-WE | 13.67 | 82 | Ningxia-CI | 8.31 |
| 33 | Henan-PG | 13.63 | 83 | Hunan-MP | 8.14 |
| 34 | Xinjiang-EP | 13.29 | 84 | Anhui-CD | 8.00 |
| 35 | Jiangxi-TW | 13.25 | 85 | Qinghai-CI | 7.98 |
| 36 | Zhejiang-MM | 13.18 | 86 | Ningxia-MP | 7.86 |
| 37 | Xinjiang-MP | 12.80 | 87 | Sichuan-TW | 7.66 |
| 38 | Hebei-EP | 12.77 | 88 | Hubei-MP | 7.61 |
| 39 | Gansu-PR | 12.76 | 89 | Ningxia-MM | 7.46 |
| 40 | Jiangsu-EP | 12.40 | 90 | Beijing-TW | 7.45 |
| 41 | Hebei-CD | 12.31 | 91 | Gansu-CI | 7.38 |
| 42 | Xinjiang-PR | 12.12 | 92 | Xinjiang-WE | 7.19 |
| 43 | Ningxia-WE | 12.08 | 93 | Sichuan-PG | 7.11 |
| 44 | Inner Mongolia-WE | 12.01 | 94 | Heilongjiang-EP | 7.08 |
| 45 | Zhejiang-EP | 12.01 | 95 | Jiangsu-CD | 6.93 |
| 46 | Henan-EP | 11.98 | 96 | Shanghai-OP | 6.82 |
| 47 | Fujian-EP | 11.86 | 97 | Guangxi-TW | 6.49 |
| 48 | Jilin-EP | 11.83 | 98 | Xinjiang-CI | 6.38 |
| 49 | Xinjiang-GP | 11.77 | 99 | Henan-GP | 6.37 |
| 50 | Guizhou-EP | 11.70 | 100 | Heilongjiang-TW | 6.37 |

**Note:** Letters following regions denote sectors (see Table S2 for the full names of sectors).

# Table S6. Top 100 region sectors with the highest VI in 2017

| **Rank** | **region-sector** | **VI**  **(10^-3^)** | **Rank** | **region-sector** | **VI**  **(10^-3^)** |
| --- | --- | --- | --- | --- | --- |
| 1 | Hainan-MR | 62.28 | 51 | Henan-MR | 24.41 |
| 2 | Tibet-MP | 56.25 | 52 | Inner Mongolia-GE | 24.38 |
| 3 | Chongqing-WE | 55.73 | 53 | Chongqing-EP | 24.22 |
| 4 | Tibet-EP | 52.99 | 54 | Jilin-MP | 24.08 |
| 5 | Guangdong-EP | 51.86 | 55 | Ningxia-SE | 24.02 |
| 6 | Tibet-MR | 49.22 | 56 | Ningxia-PR | 23.62 |
| 7 | Guangdong-MP | 48.05 | 57 | Tibet-WT | 23.54 |
| 8 | Zhejiang-MP | 46.67 | 58 | Guizhou-SE | 23.36 |
| 9 | Jilin-WE | 46.66 | 59 | Guangdong-EE | 23.16 |
| 10 | Hainan-EE | 46.63 | 60 | Jilin-GE | 23.01 |
| 11 | Chongqing-MP | 46.07 | 61 | Beijing-MR | 22.95 |
| 12 | Shaanxi-MR | 41.86 | 62 | Chongqing-TE | 22.72 |
| 13 | Heilongjiang-MR | 40.17 | 63 | Guangdong-GE | 22.63 |
| 14 | Guangdong-MR | 36.67 | 64 | Shaanxi-PP | 22.43 |
| 15 | Chongqing-EE | 36.48 | 65 | Jiangxi-MR | 22.24 |
| 16 | Guizhou-WE | 36.22 | 66 | Shaanxi-WE | 22.23 |
| 17 | Chongqing-MR | 35.72 | 67 | Shaanxi-SR | 22.10 |
| 18 | Zhejiang-MR | 34.72 | 68 | Ningxia-EP | 22.03 |
| 19 | Jilin-MR | 34.32 | 69 | Yunnan-WE | 21.92 |
| 20 | Henan-EP | 33.58 | 70 | Anhui-MR | 21.61 |
| 21 | Shaanxi-IM | 32.03 | 71 | Ningxia-MR | 21.50 |
| 22 | Tibet-EE | 32.02 | 72 | Guizhou-EE | 21.42 |
| 23 | Beijing-MP | 31.53 | 73 | Heilongjiang-SE | 21.38 |
| 24 | Hunan-EP | 31.30 | 74 | Xinjiang-MR | 21.24 |
| 25 | Hebei-EP | 30.72 | 75 | Yunnan-MR | 21.19 |
| 26 | Guangxi-EP | 30.61 | 76 | Inner Mongolia-MR | 21.12 |
| 27 | Chongqing-WR | 30.33 | 77 | Jilin-WR | 21.07 |
| 28 | Hainan-SE | 30.07 | 78 | Guizhou-GE | 21.03 |
| 29 | Hainan-WR | 29.32 | 79 | Shaanxi-WR | 21.01 |
| 30 | Zhejiang-GE | 28.73 | 80 | Hainan-TE | 20.90 |
| 31 | Hainan-GE | 28.23 | 81 | Guizhou-TE | 20.73 |
| 32 | Zhejiang-EE | 27.98 | 82 | Shaanxi-MP | 20.68 |
| 33 | Qinghai-MR | 27.82 | 83 | Guangdong-SE | 20.64 |
| 34 | Henan-GP | 27.58 | 84 | Guizhou-MR | 20.52 |
| 35 | Heilongjiang-WE | 27.45 | 85 | Guizhou-WR | 20.49 |
| 36 | Shaanxi-SE | 27.40 | 86 | Xinjiang-EE | 20.43 |
| 37 | Chongqing-GE | 26.48 | 87 | Hunan-PR | 20.38 |
| 38 | Tibet-WR | 26.42 | 88 | Anhui-MP | 20.31 |
| 39 | Jilin-SE | 26.10 | 89 | Shaanxi-PA | 20.04 |
| 40 | Zhejiang-SE | 26.06 | 90 | Heilongjiang-MP | 20.03 |
| 41 | Jiangxi-EP | 25.99 | 91 | Xinjiang-GE | 19.80 |
| 42 | Jilin-OP | 25.98 | 92 | Henan-EE | 19.48 |
| 43 | Zhejiang-WR | 25.70 | 93 | Zhejiang-EP | 19.47 |
| 44 | Heilongjiang-EE | 25.65 | 94 | Guangdong-WE | 19.31 |
| 45 | Shaanxi-TE | 25.44 | 95 | Yunnan-GE | 19.05 |
| 46 | Anhui-EP | 25.33 | 96 | Tibet-SE | 18.95 |
| 47 | Shaanxi-GE | 25.15 | 97 | Jiangxi-PR | 18.92 |
| 48 | Ningxia-EE | 24.66 | 98 | Inner Mongolia-SE | 18.73 |
| 49 | Yunnan-EE | 24.65 | 99 | Chongqing-SE | 18.70 |
| 50 | Heilongjiang-GE | 24.48 | 100 | Hainan-PM | 18.67 |

**Note:** Letters following regions denote sectors (see Table S2 for the full names of sectors).

# Table S7. Top 100 region-sectors with increased VESR exports from 2012 to 2017

| **Rank** | **region-sector** | **Increased value** | **Rank** | **region-sector** | **Increased value** |
| --- | --- | --- | --- | --- | --- |
| 1 | Shanghai-MP | 51.29 | 51 | Gansu-CD | 0.83 |
| 2 | Jiangsu-MP | 48.05 | 52 | Tianjin-TW | 0.80 |
| 3 | Inner Mongolia-MP | 23.88 | 53 | Hainan-PP | 0.79 |
| 4 | Fujian-MP | 21.07 | 54 | Tianjin-EP | 0.76 |
| 5 | Shandong-CD | 17.59 | 55 | Inner Mongolia-AS | 0.76 |
| 6 | Sichuan-TW | 15.05 | 56 | Yunnan-WT | 0.74 |
| 7 | Xinjiang-MP | 12.92 | 57 | Guizhou-RE | 0.67 |
| 8 | Xinjiang-PG | 11.76 | 58 | Anhui-WE | 0.66 |
| 9 | Shanghai-PR | 10.19 | 59 | Gansu-NP | 0.63 |
| 10 | Zhejiang-TW | 8.75 | 60 | Hebei-ET | 0.60 |
| 11 | Heilongjiang-WT | 8.14 | 61 | Hebei-PM | 0.57 |
| 12 | Guizhou-WT | 7.86 | 62 | Heilongjiang-TW | 0.53 |
| 13 | Hainan-CI | 7.26 | 63 | Shanghai-CI | 0.52 |
| 14 | Beijing-EP | 6.48 | 64 | Jilin-WT | 0.49 |
| 15 | Shanghai-ET | 6.46 | 65 | Heilongjiang-PG | 0.47 |
| 16 | Guangxi-NP | 6.44 | 66 | Xinjiang-NP | 0.44 |
| 17 | Hubei-TW | 6.21 | 67 | Jiangsu-CI | 0.43 |
| 18 | Qinghai-PG | 5.54 | 68 | Hebei-WT | 0.42 |
| 19 | Jilin-TW | 5.42 | 69 | Xinjiang-PR | 0.40 |
| 20 | Shandong-PG | 5.41 | 70 | Guangdong-EQ | 0.37 |
| 21 | Yunnan-CD | 4.64 | 71 | Qinghai-WT | 0.37 |
| 22 | Guizhou-MP | 4.60 | 72 | Guizhou-FS | 0.36 |
| 23 | Gansu-PR | 4.22 | 73 | Heilongjiang-SR | 0.36 |
| 24 | Jilin-MP | 3.96 | 74 | Zhejiang-NP | 0.33 |
| 25 | Guangdong-MP | 3.82 | 75 | Guangxi-PR | 0.33 |
| 26 | Jiangxi-MP | 3.81 | 76 | Guangdong-PM | 0.33 |
| 27 | Guangxi-MP | 3.71 | 77 | Zhejiang-PR | 0.32 |
| 28 | Chongqing-CI | 3.64 | 78 | Yunnan-WE | 0.32 |
| 29 | Fujian-PR | 3.30 | 79 | Tianjin-MR | 0.29 |
| 30 | Sichuan-PG | 3.22 | 80 | Heilongjiang-AS | 0.29 |
| 31 | Xinjiang-CD | 3.15 | 81 | Anhui-PM | 0.29 |
| 32 | Shanghai-EP | 3.02 | 82 | Guangdong-PP | 0.28 |
| 33 | Henan-CD | 3.02 | 83 | Hunan-PM | 0.28 |
| 34 | Shaanxi-CD | 2.83 | 84 | Guangdong-CI | 0.27 |
| 35 | Ningxia-CI | 2.55 | 85 | Chongqing-TW | 0.27 |
| 36 | Inner Mongolia-PR | 2.31 | 86 | Guizhou-ET | 0.27 |
| 37 | Jiangxi-NP | 1.85 | 87 | Shanghai-IS | 0.25 |
| 38 | Hebei-PG | 1.78 | 88 | Zhejiang-MM | 0.25 |
| 39 | Heilongjiang-CD | 1.51 | 89 | Hebei-RE | 0.24 |
| 40 | Hubei-MP | 1.50 | 90 | Shanghai-RE | 0.23 |
| 41 | Liaoning-PR | 1.34 | 91 | Inner Mongolia-MM | 0.23 |
| 42 | Hainan-MM | 1.18 | 92 | Shanghai-WT | 0.23 |
| 43 | Xinjiang-CI | 1.11 | 93 | Guangxi-PM | 0.23 |
| 44 | Qinghai-MP | 1.11 | 94 | Guangdong-ET | 0.22 |
| 45 | Hunan-WT | 1.08 | 95 | Zhejiang-RE | 0.22 |
| 46 | Heilongjiang-NP | 1.07 | 96 | Jiangxi-WT | 0.22 |
| 47 | Shanghai-PM | 1.02 | 97 | Henan-PG | 0.21 |
| 48 | Inner Mongolia-CD | 1.00 | 98 | Jiangsu-MM | 0.21 |
| 49 | Jiangsu-CD | 0.95 | 99 | Xinjiang-AS | 0.20 |
| 50 | Liaoning-MM | 0.87 | 100 | Shanghai-CN | 0.20 |

**Note:** Letters following regions denote sectors (see Table S2 for the full names of sectors).

# Table S8. Top 100 region-sectors with increased VESR imports from 2012 to 2017

| **Rank** | **region-sector** | **Increased value** | **Rank** | **region-sector** | **Increased value** |
| --- | --- | --- | --- | --- | --- |
| 1 | Zhejiang-WR | 34.25 | 51 | Shaanxi-SR | 2.17 |
| 2 | Chongqing-WR | 26.89 | 52 | Gansu-WR | 2.13 |
| 3 | Shaanxi-WR | 16.96 | 53 | Qinghai-WR | 2.00 |
| 4 | Jiangsu-WR | 16.90 | 54 | Shandong-IS | 1.92 |
| 5 | Yunnan-WR | 16.07 | 55 | Guangdong-SE | 1.83 |
| 6 | Guangdong-WR | 14.46 | 56 | Henan-IS | 1.82 |
| 7 | Henan-WR | 11.86 | 57 | Jiangxi-ER | 1.79 |
| 8 | Beijing-WR | 11.23 | 58 | Henan-NP | 1.75 |
| 9 | Liaoning-WR | 11.18 | 59 | Hebei-ER | 1.70 |
| 10 | Jiangxi-WR | 10.30 | 60 | Hebei-WT | 1.69 |
| 11 | Guizhou-WR | 10.25 | 61 | Fujian-WT | 1.62 |
| 12 | Sichuan-WR | 9.66 | 62 | Tianjin-RE | 1.48 |
| 13 | Anhui-WR | 9.31 | 63 | Henan-FM | 1.45 |
| 14 | Inner Mongolia-WR | 9.03 | 64 | Hunan-ER | 1.42 |
| 15 | Hunan-WR | 8.34 | 65 | Shaanxi-PA | 1.37 |
| 16 | Henan-WT | 7.48 | 66 | Anhui-ER | 1.36 |
| 17 | Xinjiang-WR | 7.37 | 67 | Guizhou-WT | 1.32 |
| 18 | Jilin-WR | 7.18 | 68 | Chongqing-RE | 1.30 |
| 19 | Hebei-WR | 6.78 | 69 | Jiangsu-WT | 1.30 |
| 20 | Shanghai-WR | 6.55 | 70 | Yunnan-RE | 1.28 |
| 21 | Zhejiang-ER | 6.37 | 71 | Guangdong-GE | 1.23 |
| 22 | Shanxi-WR | 6.28 | 72 | Guangxi-IS | 1.13 |
| 23 | Guangxi-WR | 6.12 | 73 | Jiangxi-EE | 1.13 |
| 24 | Hainan-WR | 5.99 | 74 | Tianjin-ER | 1.09 |
| 25 | Beijing-RE | 5.37 | 75 | Fujian-RE | 1.09 |
| 26 | Heilongjiang-WR | 5.10 | 76 | Zhejiang-WT | 1.08 |
| 27 | Tianjin-WR | 5.00 | 77 | Chongqing-EQ | 1.01 |
| 28 | Shandong-WR | 4.86 | 78 | Jilin-MR | 0.98 |
| 29 | Jiangsu-ER | 4.84 | 79 | Shaanxi-PP | 0.98 |
| 30 | Henan-MR | 4.45 | 80 | Chongqing-WT | 0.94 |
| 31 | Guangdong-ER | 4.45 | 81 | Anhui-EQ | 0.92 |
| 32 | Guangdong-MP | 4.25 | 82 | Henan-EQ | 0.92 |
| 33 | Beijing-ER | 3.87 | 83 | Xinjiang-ER | 0.91 |
| 34 | Anhui-WT | 3.66 | 84 | Guizhou-RE | 0.89 |
| 35 | Tibet-WR | 3.63 | 85 | Henan-OS | 0.89 |
| 36 | Beijing-HR | 3.54 | 86 | Liaoning-WT | 0.87 |
| 37 | Jiangsu-IS | 3.54 | 87 | Inner Mongolia-WT | 0.87 |
| 38 | Jiangsu-RE | 3.23 | 88 | Sichuan-TE | 0.86 |
| 39 | Guangdong-MR | 3.21 | 89 | Guangxi-WT | 0.86 |
| 40 | Shanghai-RE | 2.88 | 90 | Shanghai-ER | 0.85 |
| 41 | Zhejiang-RE | 2.85 | 91 | Guizhou-ER | 0.82 |
| 42 | Henan-EE | 2.82 | 92 | Heilongjiang-ER | 0.81 |
| 43 | Ningxia-WR | 2.61 | 93 | Xinjiang-WT | 0.79 |
| 44 | Guangdong-RE | 2.58 | 94 | Shaanxi-RE | 0.79 |
| 45 | Shandong-RE | 2.51 | 95 | Qinghai-MP | 0.79 |
| 46 | Fujian-WR | 2.50 | 96 | Liaoning-ER | 0.79 |
| 47 | Hubei-WR | 2.47 | 97 | Jilin-ER | 0.78 |
| 48 | Anhui-RE | 2.34 | 98 | Shaanxi-ER | 0.74 |
| 49 | Tianjin-WT | 2.29 | 99 | Guangxi-ER | 0.73 |
| 50 | Henan-ER | 2.24 | 100 | Hebei-OS | 0.72 |

**Note:** Letters following regions denote sectors (see Table S2 for the full names of sectors).
